# Exploring the Entropy Complex Networks with Latent Interaction

**DOI:** 10.3390/e25111535

**Published:** 2023-11-11

**Authors:** Alex Arturo Centeno Mejia, Moisés Felipe Bravo Gaete

**Affiliations:** 1 Doctorado en Modelamiento Matemático Aplicado, Universidad Católica del Maule, Avenida San Miguel, Talca 3605, Chile; 2 Departamento de Matemáticas, Física y Estadística, Facultad de Ciencias Básicas, Universidad Católica del Maule, Avenida San Miguel, Talca 3605, Chile; mbravo@ucm.cl

**Keywords:** entropy, complex networks, latent interaction index, estimation

## Abstract

In the present work, we study the introduction of a latent interaction index, examining its impact on the formation and development of complex networks. This index takes into account both observed and unobserved heterogeneity per node in order to overcome the limitations of traditional compositional similarity indices, particularly when dealing with large networks comprising numerous nodes. In this way, it effectively captures specific information about participating nodes while mitigating estimation problems based on network structures. Furthermore, we develop a Shannon-type entropy function to characterize the density of networks and establish optimal bounds for this estimation by leveraging the network topology. Additionally, we demonstrate some asymptotic properties of pointwise estimation using this function. Through this approach, we analyze the compositional structural dynamics, providing valuable insights into the complex interactions within the network. Our proposed method offers a promising tool for studying and understanding the intricate relationships within complex networks and their implications under parameter specification. We perform simulations and comparisons with the formation of Erdös–Rényi and Barabási–Alber-type networks and Erdös–Rényi and Shannon-type entropy. Finally, we apply our models to the detection of microbial communities.

## 1. Introduction

Complex Network Analysis (CNA) is a crucial field that spans various disciplines, addressing network dynamics [1,2,3]. In this context, the focus is on unraveling the complexities of network structure, specifically in the dynamics of link formation. The study delves into three fundamental network attributes: the homophily effect, unobserved heterogeneity, and persistence measures. Homophily, which denotes the tendency of nodes to connect with similar nodes, is a well-established phenomenon in real-world social networks [4]. However, many node characteristics influencing linking decisions remain unobservable, outnumbering observable ones. To address this challenge, a fixed effect approach to account for unobserved heterogeneity is introduced [5,6,7], as well as persistence measures as tools for quantifying time series data dependence [8]. These measures hold significant implications for various processes, for example, in information diffusion and ecological networks (see refs. [1,9,10,11]).

While complex networks offer powerful modeling capabilities, they also present significant challenges. One major hurdle is the lack of a comprehensive metric to effectively measure unobserved heterogeneity, especially in understanding interconnected components [12]. This metric should consider interaction abundance and latent nature, aligning with existing frameworks [13,14]. Furthermore, metrics assessing heterogeneity, both observed and unobserved, are intricately linked to the ways in which nodes are aggregated. From the above, an acute dilemma arises when unobserved heterogeneity is treated as an incidental parameter, independent of node aggregation. In such cases, the parameter vector dimension grows with network size, leading to non-standard estimation challenges, where classical results regarding the properties of maximum likelihood estimates (MLEs) no longer apply [15]. Additionally, certain models (see, for example, refs. [7,16,17]) disregard interdependencies in network formation. These limitations prompt essential questions: Can we devise a test for evaluating the link formation interdependence hypothesis? Is it feasible to extend the model’s scope to incorporate these interdependencies? How can we address the challenges posed by complex network structures and their inherent uncertainties for a deeper understanding of link formation dynamics?

In this work, we introduce a novel framework to tackle these complex network analysis challenges, where our approach (i) incorporates a discrete latent interaction index that integrates parametric and semiparametric components, shedding light on network formation dynamics.

The realm of network models is diverse, ranging from classical ones [18,19,20] to the more recent advancements [6,7,16]. In the first group of models (I), random networks [18] aim to probabilistically study graph properties as the number of random connections increases, reflecting the disordered nature of link arrangements between different nodes. We start with the hypothesis that the proposed latent interaction index displaces the possibility of randomness in link formation. We conduct statistical significance tests based on this hypothesis. Additionally, the Watts–Strogatz model [20] presents a rewiring model that often exhibits high clustering coefficients in “small” networks. On the other hand, the Barabási–Albert model [19] relies on two ingredients: growth and preferential attachment. The idea is that by mimicking the dynamic mechanisms that assemble the network, we can reproduce the system’s topological properties as observed here. The second group of models (II) has been limited to studying static nonlinear dyadic models and their asymptotic properties. Because the number of individual parameters is proportional to the number of nodes, a problem of incidental parameters results in asymptotic bias [6]. While the estimator is consistent, asymptotic bias is relevant for inference. We provide a model test based on the prevalence of transitive triads (i.e., node triples where links are transitive). Observed heterogeneity has also been incorporated through dyadic models that expand on this model, just as a probit or logit model generalizes a simple Bernoulli statistical model, which can be used in directed or undirected settings [21]. It is possible to extend the Erdös–Rényi model to incorporate other features [5].

Our proposed model seeks to bridge these two groups (I and II), offering a comprehensive approach to network analysis by incorporating the strengths of both.

Additionally, (ii) we present an entropy function dynamically accounting for these components, providing insights into parameters related to persistence and homophily. The estimates derived from this entropy function provide valuable information to characterize the parameters related. This framework enhances our comprehension of link formation within dynamic networks, enabling us to explore the influence of these components on network formation and evolution [22,23,24].

To provide a comprehensive view, it is important to note that each entropy metric used in network analysis offers unique insights into network characteristics and its various components. However, it is widely acknowledged within the field that not all of these metrics can be universally applied to all categories of networks. In fact, this wealth of research is dispersed across numerous disciplines [1,17,22,23,25,26], making it challenging to identify the available metrics and understand the specific contexts in which they are applicable. Additionally, this dispersion complicates our ability to determine areas in need of further development.

These entropy metrics often depend on probability distributions based on various factors, such as node degrees [27,28], the degree and strength of node neighbors [23,29,30], or degrees associated with subgraphs of nodes [31]. Path-based metrics, considering sequences of linked nodes and repetitions of nodes and edges, are also common [32,33,34]. Moreover, entropy metrics explore other factors like closeness and information functionals [35,36]. Some metrics rely on probability distribution, including Bayes posterior probability, although specific calculation methods may not always be clear [37]. Notably, Wang et al. [38] introduced a combined metric, where the first part is calculated as the sum of closeness centrality and the clustering coefficient.

Ecological research has a long-standing tradition of studying co-occurrence and co-abundance patterns. These patterns often signify non-random species co-occurrence, indicating that interactions play a significant role in community structure—either by fostering aggregation or promoting avoidance/exclusion—thus influencing the overall community dynamics. Macro-ecological interaction networks illustrate that such patterns bolster community robustness and functionality, crucial for comprehending community dynamics and productivity [25,39]. Microorganisms engage in diverse relationships, encompassing both antagonistic and cooperative interactions. With the advancements in sequencing technologies, we now have access to substantial datasets for analysis. This allows for the construction of co-occurrence networks using correlation coefficients or similar metrics. However, interpreting these networks, especially in microbial surveys with poorly understood organism behaviors, presents significant challenges [11,17,40].

The complexity of microbial communities makes it challenging to validate community-wide interactions due to the multitude of species and limited experimental approaches. Consequently, modeling microbial populations using simplified growth and interaction rules offers an alternative approach to simulate the dynamics of these intricate multispecies communities. In this study, we consider the model proposal as an application for identifying microbial networks. Concretely, we apply our dynamic network formation model on an 18S rRNA gene amplicon dataset. The original dataset comprises 19 samples, and we observe a total of 3831 OTU (Operative Taxonomic Unity) entries. These observations are obtained through Lagrangian sampling as part of a study conducted by Hu et al. [40].

This work starts by providing an introduction to our notations, delineating the symbols and conventions used throughout this study. The organization of this paper is as follows: In Section 2, we present the proposed model; then, in Section 3, we introduce the entropy function. In Section 4, simulation results are presented, and in Section 5 we apply the model focused on the microbial network identification. Section 6 provides the conclusions and discussions, while all proofs of the theorems and elimination of fixed effects are present in Appendix A.

**Notation** **1.** *Network G=(V,E) is an ordered pair of sets V and E, where V is a set finite nonempty of elements named nodes, and the set E is composed of two-element subsets {ij} of V named edges. If i and j are connected, {ij} constitutes a dyad, and j is a neighbor of i. Along the work, we use notation $\prod_{i<j}$ to indicate $\prod_{i=1}^{N}\prod_{j=i+1}^{N}$, and similarly $\sum_{i<j}$ to indicate $\sum_{i=1}^{N-1}\sum_{j=i+1}^{N}$.*

## 2. Structural Model

We consider a dynamic group interaction scenario consisting of a large population of connected nodes. We let i=1,…,N is the index of a random sample of size *N* from this population at time t=1,…,T. Each node *i* has a profile defined as ${\left(X_{i,t}^{⊤},A_{i}\right)}^{⊤}$, where $X_{i,t}$ is an aggregated vector of the observed time-varying characteristics, $A_{i}$ contains unobserved information assuming the *t*-invariant. We let $\mathrm{Supp}(X_{i,t})$ be a compact subset of $R^{\dim(X_{i,t})}$, and $A_{i}$ is distributed compactly and continuously on the same support, conditional on $X_{i,t}=x$, i.e., for all $x\in \mathrm{Supp}(X_{i,t})$, $\mathrm{Supp}\left(A_{i}|X_{i,t}=x\right)=\mathrm{Supp}\left(A_{i}\right)$ is a compact subset of R.

Linking decisions are a binary choice that depends solely on the characteristics of the two nodes connected by the link. We observe relationships between nodes through the indicator variable $C_{ij,t}∼\mathrm{Bernoulli}\left(p_{ij,t}\right)$, where $C_{ij,t}=1$ if node *j* interacts (success) with node *i* at time *t* and $C_{ij,t}=0$ (failure) otherwise. Parameter $p_{ij,t}$ can be interpreted as the detection rate of the interaction between nodes *i*, *j*. Connections are undirected (i.e., $C_{ij,t}=C_{ji,t}$), and self-ties are ruled out (i.e., $C_{ii,t}=0$ for all *t*). For each t=1,…,T, there is a corresponding N×N socio-matrix $C={\left(C_{ij,t}\right)}_{i\neq j}$ that captures the interaction dynamics between nodes *i* and *j* across all time steps.

We parameterize the latent interaction structure according to the probability of each link $C_{ij,t}$:

(1) $$C_{ij,t}=1\left\{\sum_{p=1}^{q}\alpha_{p}^{0}C_{ij,t-p}+\beta^{0}X_{ij,t}^{⊤}+D_{ij,t}+A_{ij}-\epsilon_{ij,t}>0\right\},$$

where **1**(·) denotes the indicator function. The *q*-dimensional vector $\alpha^{0}={\left(\alpha_{1}^{0},\cdots ,\alpha_{q}^{0}\right)}^{⊤}$ with ∥α∥ < 1 and 1≤q≤t−1 captures the autocorrelation or cumulative nonlinear persistence of the time series [8]. Variable $X_{ij,t}:\mathrm{Supp}\left(X_{i,t}\right)\times \mathrm{Supp}\left(X_{j,t}\right)\to R^{\dim(\beta )}$ is a known transformation of ${\left(X_{i,t}^{⊤},X_{j,t}^{⊤}\right)}^{⊤}$. This function is symmetric, so that $X_{ij,t}=X_{ji,t}$. For example, if $X_{i,t}$ and $X_{jt}$ are location coordinates, $X_{ij,t}$ is equal to the “distance” between *i* and *j*. This choice was implemented under the consideration that nodes only form connections if they are close enough [7,16,21]. Vector $\beta^{0}$ is an unknown model parameter that parameterizes homophily preferences. The parameter vector is $\theta^{0}={\left({\left(\alpha^{0}\right)}^{⊤},{\left(\beta^{0}\right)}^{⊤}\right)}^{⊤}\in \mathrm{int}\left(\Theta \right)$, with Θ being a compact subset of $R^{q+\dim(\beta )}$. Variable $D_{ij,t}=\sum_{t^{\prime }\leq t}\sum_{k=1}^{N}C_{ik,t^{\prime }}C_{jk,t^{\prime }}$ denotes the memory effect of connections that node *i* and *j* have had in common up to time *t*. Variable $A_{ij}$ is a component that varies with unobserved attributes by node pairs as in Graham’s model [7], and $\epsilon_{ij,t}$ represent an idiosyncratic component that is assumed to be independent and identically distributed over time. Moreover, this component is assumed to be independent across pairs, although not necessarily identically distributed; it is

(2) $$F\left(\epsilon_{12,1},\ldots ,\epsilon_{12,T},\ldots ,\epsilon_{(N-1)N,1},\ldots ,\epsilon_{(N-1)N,T}\right)=\prod_{i<j}\prod_{t=1}^{T}F_{\epsilon_{ij,t}}.$$

It is important to note that Equation (1) captures in a parsimonious way three forces that researchers consider important for bond formation [41]. First, linkages are state dependent; equally, the linkage returns for *i* and *j* are higher in the current period if they were also connected in previous periods. Second, there are returns to “triadic closure”, profit is higher if transitive aspects are considered in the interaction between nodes. In addition, Rule (1) is more general instead of taking $D_{ij,t}=0$ and $\alpha_{p}^{0}=0$ for all p=1,…,q, which would imply that only direct entailments are important, not autocorrelation and particular incentives for interaction.

The degree of a node is defined as the number of links it possesses, which can be represented as the sum of connections it has with other nodes, and denoted as $C_{i+,t}=\sum_{i\neq j}C_{ij,t}$. The network’s degree sequence is obtained by summing the rows (or columns) of the adjacency matrix, resulting in an N×1 vector $C_{+}={\left(C_{1+,t},\ldots ,C_{N+,t}\right)}^{⊤}$.

We denote

$$z_{ij,t}\left(\theta ,A_{ij}\right)=\exp\left(\sum_{p=1}^{q}\alpha_{p}C_{ij,t-p}+\beta X_{ij,t}^{⊤}+D_{ij,t}+A_{ij}\right).$$

For parameter values θ∈int(Θ) and $A={\left({\left(A_{i}\right)}_{i=1,\ldots ,N},{\left(A_{j}\right)}_{j=1,\ldots ,N}\right)}^{⊤}\in \mathrm{Supp}\left(A\right)$, we define the link probability $p_{ij,t}\left(\theta ,A_{ij}\right)=\frac{z_{ij,t}\left(\theta ,A_{ij}\right)}{1+z_{ij,t}\left(\theta ,A_{ij}\right)}$.

With the information presented above, we are now able to outline the principal assumptions that significantly influence our work:

**Assumption** **1.** *Equations *(1)* and *(2)* specify a dynamic model of node interactions. The conditional likelihood of link $C_{ij,t}=c_{ij,t}$ is given by*

(3) $$\mathrm{Pr}\left(C_{ij,t}=c_{ij,t}|X,D,A_{0}\right)=\prod_{i<j}\prod_{t=1}^{T}\frac{z_{ij,t}^{c_{ij,t}}\left(\theta^{0},A_{ij_{0}}\right)}{1+z_{ij,t}\left(\theta^{0},A_{ij_{0}}\right)},$$

Here, Assumption 1 implies that the idiosyncratic component of link surplus, $\epsilon_{ij,t}$, is a standard logistic random variable that is independently and identically distributed across pairs of nodes. The assumption that links are formed independently of each other based on agent attributes may hold in some situations but not in others. Specifically, Equation (1) and Assumption 1 are suitable for scenarios where link formation is predominantly bilateral. This is particularly relevant in certain types of friendship and trade networks, as well as in models of specific types of conflicts between nation-states [42,43]. In these contexts, the incorporation of unobserved node characteristics into the link formation model represents a significant and useful generalization relative to many commonly used models.

The objective pursued here is to study the identification and estimation problems posed by the shape according to Equation (1) and Assumption 1. This set encompasses a useful class of empirical examples and represents a natural starting point for a formal statistical analysis. In this context, early methodological work focused on introducing unobserved correlated heterogeneity into static choice models [44,45]. Subsequent work incorporated a chance for stated dependence in choice [46].

The estimated value of the parameters, denoted by

(4) $$\begin{array}{ccc} \hat{\theta } & = & {\left({\hat{\alpha }}^{⊤},{\hat{\beta }}^{⊤}\right)}^{⊤}, \end{array}$$

(5) $$\begin{array}{ccc} {\hat{a}}_{N} & = & {\left({\hat{A}}_{1},\ldots ,{\hat{A}}_{N}\right)}^{⊤}, \end{array}$$

are the solution to the population conditional maximum likelihood problem

(6) $$\begin{array}{c} \max_{\left(\theta ,a_{N}\right)\in R^{\dim(\theta )+N}}E_{a}\left[L_{NT}\left(\theta ,a_{N}\right)\right],\\ L_{NT}\left(\theta ,a_{N}\right):={\left(NT\right)}^{-\frac{1}{2}}\left\{\sum_{i<j}\sum_{t=1}^{T}C_{ij,t}\log\left(p_{ij,t}\left(\theta ,A_{ij}\right)\right)+\left(1-C_{ij,t}\right)\log\left(1-p_{ij,t}\left(\theta ,A_{ij}\right)\right)\right\}, \end{array}$$

for every N,T. Here, $E_{a}$ denotes the expectation with respect to the distribution of the data conditional on the unobserved effects.

**Assumption** **2.**

(*i*) *Asymptotics: We consider limits of sequences where N/T approaches a constant value c as both N and T rise to infinity, where c is a finite number greater than zero.*
(*ii*) *Sampling: Conditional on $a_{N}={\left(A_{1},\ldots ,A_{N}\right)}^{⊤}$, $\left\{(C_{ij,t},X_{ij,t}):1\leq i,j\leq N,1\leq t\leq T\right\}$ is independent across the dyad, and for $Y_{ij,t}=\left(C_{ij,t},X_{ij,t}\right)$, A is the σ-field generated by $(Y_{ij,t},$$Y_{ij,t-1}{,\ldots )}^{⊤}$, and B is the σ-field generated by ${\left(Y_{ij,t},Y_{ij,t+1,\ldots }\right)}^{⊤}$.*
(*iii*) *Compact support: The support of $X_{ij,t}$ is a compact subset of $R^{\dim(\beta )}$.*
(*iv*) *Concavity: For all N,T, $\left(\theta ,a_{N}\right)\mapsto L_{NT}\left(\theta ,a_{N}\right)$ is strictly concave over $R^{\dim\theta +N}$.*

Just for completeness, Assumption 2 (i) defines the large-*T* asymptotic framework and is the same as in Hahn and Kuersteiner [47]. The relative rate exactly balances the order of the bias and variance producing a non-degenerate asymptotic distribution. Assumption 2 (ii) imposes neither identical distribution nor stationarity over the time series dimension, conditional on the unobserved effects, unlike most of the large-*T* panel literature [47]. Additionally, it is used to bound covariances and moments in the application of the Laws of Large Numbers (LLN), as we see below, it could be replaced by other conditions that guarantee the applicability of these results. Assumption 2 (iii) is standard in the context of nonlinear estimation problems [48]. It implies that the observed component of link surplus, $\sum_{p=1}^{q}\alpha_{p}c_{ij,t-p}+\beta x_{ij,t}^{⊤}+d_{ij,t}$, has bounded support. This simplifies the proofs of the main theorems, especially those of the ML estimator. Furthermore, (iv) imposes smoothness and moment conditions in the log-likelihood function and its derivatives. These conditions guarantee that the higher-order stochastic expansions of the fixed effect estimator that we use to characterize the asymptotic bias are well-defined, and the remaining terms of these expansions are bounded. In addition, this guarantees that all the elements of $X_{ij,t}$ have cross-sectional and time series variation. In addition, it also guarantees that $\hat{\theta }$ is the unique solution to the population problem (given by Equation (6)), that is, all the parameters are point identified. The existence and uniqueness of the solution to the population problem are guaranteed by our Assumptions 2, including the concavity of the objective function in all parameters.

Together with the above, and denoting $p_{ij,t}=p_{ij,t}\left(\theta^{0},a_{N}^{0}\right)$, through to Parts (iii) and (iv) from Assumption 2 in combination with $\mathrm{Supp}(A_{i})$ being a compact subset of R, our findings imply that $p_{ij,t}\left(\theta ,a_{N}\right)\in \left(\kappa ,1-\kappa \right)$ for some 0<κ<1 and for all θ and $a_{N}\in \mathrm{Supp}\left(A\right)$. An implication of this fact is that $\left(C_{ij,t}-p_{ij,t}\right)\log\left(p_{ij,t}\left(\theta ,a_{N}\right)\right)$ is a bounded random variable. A more involved argument shows that it is possible to estimate the difference between $C_{ij,t}$ and $p_{ij,t}$ with uniform accuracy.

With the aforementioned assumptions in place, we can now elucidate the primary theorems that are providential through the work:

**Theorem** **1.** *Under Assumptions 1 and 2,*

(7) $$\underset{1\leq i,j\leq N}{\sup}\underset{1\leq t\leq T}{\sup}\left|\frac{1}{(N-1)T}\sum_{i<j}\sum_{t=1}^{T}\left(C_{ij,t}-p_{ij,t}\right)\right|<\sqrt{\ln(NT)}$$

*with probability $1-O\left({\left(NT\right)}^{-2}\right)$.*

Theorem 1 suggests that as more data are collected (increasing *N*) and a broader time horizon is considered (increasing *T*), the difference between latent variables and observed probabilities becomes relatively small and tends to be more bounded. This interpretation may be relevant for assessing the accuracy or validity of a latent model in relation to real observations within a network. The term ln(NT) in the upper bound can be interpreted as a measure of the uncertainty associated with the difference between latent variables and observations. As *N* and *T* grow, uncertainty decreases.

The following theorem is related to a generalized form of the Law of Large Numbers (LLN) adapted to the context of complex networks.

**Theorem** **2.** *Under Assumptions 1 and 2, we assume that*

$$E\left(\underset{\theta \in \Theta }{\sup}\left|\log\left(\frac{z_{ij,t}\left(\theta ,a_{N}\right)}{1+z_{ij,t}\left(\theta ,a_{N}\right)}\right)\right|\right)$$

*is finite for all t and $F=\{c_{ij,t^{\prime }}:t^{\prime }<t\}$ is a filtration with respect to A; then,*

Ea∑i<jlij,tθ,aN|F→PrN2−1∑i<jElogzij,t(θ0,aN0)1+zij,t(θ0,aN0)

*uniformly in θ∈Θ.*

In the LLN, the average of random variables is expected to converge to the expected value as the sample size grows. In this case, the sum of certain probability functions $l_{ij,t}^{\theta ,a_{N}}$ for all dyads in the network converges in probability towards a sum of probabilities associated with the dyads. Convergence in probability implies that as the network size (or the number of dyads) grows, the conditional expectation of the discrete choice probabilities approaches the expected value of those probabilities for all dyads. This can have significant implications in the theory of complex networks. For example, the stability of emergent patterns: if the result holds, it implies that as the network grows, emergent patterns in discrete choices may become more stable and predictable, providing a deeper understanding of collective behavior in the network [49].

## 3. Exploring the Entropy

**Figure 1:**
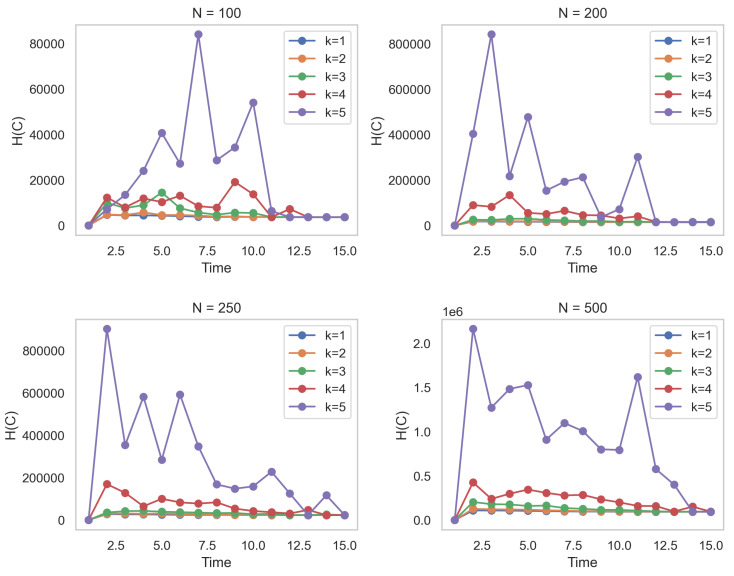
Entropy function H(C) for values of *N* nodes. Here, $\beta^{0}$ is a scalar equal to 0.5 and $\alpha^{0}$ is a random vector of length 10 with a norm of less than 1.

Combining Assumption 1 and conditional on $X_{ij,t}$, $D_{ij,t}$ and $A_{ij}$, we write

(8) $$l_{ij,t}^{\theta ,a_{N}}=c_{ij,t}\log\left(z_{ij,t}\left(\theta ,a_{N}\right)\right)-\log\left(1+z_{ij,t}\left(\theta ,a_{N}\right)\right)$$

for the log-likelihood contribution of link {ij}. Since entropy characterizes the logarithm of the number of different nodes that can be separated in the stochastic dynamics of the network [37,50], we use Equation (8) to provide a new node interaction detection rate. We note that by the asymptotic equipartition property (AEP) (see, e.g., ref. [51]), we have $-\frac{1}{TN^{2}}l_{ij,t}^{\theta ,a_{N}}$ converging in probability to the entropy of *C*, denoted as H(C), where *C* represents the socio-matrix of the network. Formally,

(9) $$H\left(C\right)=-\sum_{i<j}\sum_{t=1}^{T}l_{ij,t}^{\theta ,a_{N}}\log\left(l_{ij,t}^{\theta ,a_{N}}\right)=\sum_{i<j}\sum_{t=1}^{T}\sum_{k=1}^{+\infty }\frac{l_{ij,t}^{\theta ,a_{N}}{\left(1-l_{ij,t}^{\theta ,a_{N}}\right)}^{k}}{k},$$

where the variable *k* ranges from 1 to +*∞*, indicating that all possible configurations of connections that do not exist between nodes *i* and *j* are considered. Expression $\left[l_{ij,t}^{\theta ,a_{N}}{\left(1-l_{ij,t}^{\theta ,a_{N}}\right)}^{k}\right]/k$ represents the probability of there not being a connection between nodes *i* and *j* at time step *t*. Therefore, Equation (9) combines the influences of both existing and non-existing connections at each time step to compute the entropy of the dynamic network. For the sake of completeness, Figure 1 shows the behavior of the entropy H(C) for values of *N* nodes. It is crucial to note that Equation (9) comprehensively encompasses the charging capability of the logistics distribution—a facet that some propositions tend to disregard [52]. For the sake of completeness, Figure 1 shows the behavior of the entropy H(C) for values of *N* nodes.

The following theorems establish consistency of $\hat{\theta }$ (Equation (4)):

**Theorem** **3.** *Under Assumptions 1 and 2, we have that*

(10) $$∥\hat{\theta }-\theta^{0}∥<O\left({\left(NT\right)}^{-1/2}\right).$$

Theorem 2 provides a foundation for drawing inferences about the parameter vector encompassing homophily and nonlinear persistence. However, attaining asymptotic normality, for reasons we elaborate on, cannot be guaranteed. The consistency test for models with only individual effects is based on partitioning the log-likelihood into the sum of individual log-likelihoods that depend on a fixed number of parameters, the model parameter, and the corresponding individual effect. The individual log-likelihood maximizers are then consistent estimators of all parameters as they become large according to standard arguments. This approach does not work on network structure because there is no partition of the data that are only affected by a fixed number of parameters and whose size grows with sample size [6].

To achieve asymptotic normality over the observed, we first need to control for the unobserved and second to establish consistency in the estimated entropy function, which depends on both components.

We assess node performance and select a group of exogenous nodes to serve as a “testing ground”. To achieve this, we examine the conditional expectation of $C_{ik,t}$ and $C_{jk,t}$, conditioning on the observable characteristics of node *k*, and the characteristics of nodes *i* and *j* based on $X_{ij,t}$ and $A_{ij}$. We denote $H_{ij,t}\left(x_{k,t},a_{ij}\right)$ as the expected value of $(C_{ik,t}-C_{jk,t}$$|X_{kt}=x_{kt},A_{ij}=a_{ij},X_{ij,t}=x_{ij})$ and $\delta_{ij,t}\left(X_{k}\right)=H_{ij,t}\left(X_{k,t},A_{ij}\right)$. According to Parzen’s estimation [53] and Rosenblatt’s remarks [54], we define dyadic extension for monadic data by

(11) $${\hat{\delta }}_{ij}\left(x\right):=\frac{1}{NT}\sum_{l=1}^{N}\sum_{t=1}^{T}\left(C_{il,t}-C_{jl,t}\right)\tilde{K}\left(x-X_{l,t}\right);\;\tilde{K}\left(x-X_{l,t}\right):=\frac{K\left(\frac{x-X_{l,t}}{h(N)}\right)}{\sum_{l=1}^{N}K\left(\frac{x-X_{l,t}}{h(N)}\right)}.$$

Here, K(x) is a density function satisfying the following conditions: (i) K(x)<∞ for all *x*, (ii) symmetric around zero (K(−x)=K(x)), (iii) K(x)=0 if $|x|>\bar{x}$, and integrates to one (∫K(x)dx=1). Bandwidth h(N) is assumed to be a positive, deterministic sequence that tends to zero as N→∞.

**Lemma** **1.** *Under Assumptions 1 and 2, we have $\sup_{i,j}\left|{\hat{\delta }}_{ij}\left(x\right)-\delta_{ij}\left(x\right)\right|=O_{p}\left({\left(NT\right)}^{-1}\right)$.*

There are at least two approaches to the estimation of unobserved heterogeneity (fixed effects). The first lies in a computational perspective [6,55]. For these purposes, the solution of the (6) program for θ is the same as in the solution of the program that imposes $\iota_{N}^{⊤}a_{N}=0$ with $\iota_{N}$, a vector of *N*-ones, directly as a constraint on the optimization, which is invariant to normalization. This constrained program has good computational properties because its objective function is concave and smooth in all the parameters. The second alternative arises from Parzen’s estimations of a density function [53]. This alternative is also efficient for the estimation of unobserved heterogeneity. The problem of estimating a probability density function over the unobserved is sometimes similar to the problem of estimating maximum likelihood parameters. However, in a network setting, it is more similar to estimating the spectral density function of a stationary process [53]. Focusing on the second alternative, the following argument shows that it is possible to estimate unobserved heterogeneity with a given probability of occurrence. We consider $\iota_{\dim\theta }$ as a vector consisting of dim(θ). We let L:R→R be a Lipschitz function, differentiable, a symmetric kernel function, and $\hat{\theta }$ as in Theorem 2.

**Theorem** **4.** *Under Lemma 1, we define*

$${\hat{A}}_{l}\left(\theta \right)=\frac{1}{N}\cdot \frac{\sum_{i<j}L\left(\frac{{\hat{\delta }}_{ij}\left(x_{l}\right)}{\sigma_{N}}\right)X_{il,t}X_{jl,t}^{⊤}\theta \iota_{\dim(\theta )}^{⊤}}{\sum_{i<j}L\left(\frac{{\hat{\delta }}_{ij}\left(x_{l}\right)}{\sigma_{N}}\right)}$$

*for all l≠i,j with $\sigma_{N}$ being bandwidth. Then, $\left|{\hat{A}}_{l}\left(\hat{\theta }\right)-A_{l}\left(\theta \right)\right|=O_{p}(\max\{{\left(NT\sigma_{N}\right)}^{-1},$$\sigma_{N}{\left(NT\right)}^{-1}\})$.*

Chatterjee, Diaconis, and Sly [56] demonstrated the uniform consistency of estimator ${\hat{A}}_{l}\left(\theta \right)$ in the model that does not incorporate dyad-level covariates. The key to this theorem is the following: In sparse network sequences, we effectively witness N−1 linking decisions made by each node, which means that we observe whether node *i* links to every other node *j*. This unique feature of the problem allows for consistent estimation of ${\hat{A}}_{l}\left(\theta \right)$ for each node. The argument becomes tedious because of the interdependence of the linking decisions in the sequences of nodes *i* and *j*. However, this dependence is weak, only arising via the presence of $C_{ij,t}$ in both link sequences. Establishing asymptotic normality of $\hat{\theta }$ is also involved. This is because the sampling properties of $\hat{\theta }$ are influenced by the estimation error in ${\hat{A}}_{l}\left(\theta \right)$. This influence generates a bias in the limit distribution of $\hat{\theta }$. This bias is similar to that which arises in large *N*, large-*T* joint fixed effects estimation of non-linear panel data models [47].

To state the form of the limit distribution, we let $\hat{H}\left(C\right)$ and $H_{0}\left(C\right)$ be the entropy computed over the parameter vector $\hat{\theta }$ and $\theta^{0}$, respectively. Our objective is to estimate quantity H(C) within the family of networks C that contains nodes *i* and *j*. Our estimator is expected to provide a reliable estimate of H(C). Here, we state the following result:

**Theorem** **5.** *Under Assumptions 1 and 2,*

(12) $$\underset{C\in C}{\sup}|\hat{H}\left(C\right)-H_{0}\left(C\right)|<\frac{1}{NT}\sqrt{\log NT}$$

*with probability $1-O\left({\left(NT\right)}^{-1}\right)$.*

This inequality demonstrates that our estimator $\hat{H}\left(C\right)$ enjoys uniform consistency within class C. In simpler terms, it implies that, as our sample size *N* and time period *T* increase, the maximum absolute difference between our estimator and the true value H(C) across all sets C∈C becomes small. The probability that the bound $\frac{1}{NT}\sqrt{\log NT}$ holds is stated to be $1-O({\left(NT\right)}^{-1})$, meaning that it holds with high probability as the size of the network and the number of time steps grow large. This result provides an upper bound on the discrepancy between the estimated and true entropy, ensuring the reliability of the estimation in the context of the class of networks C.

Now, via definition

(13) J0(θ)=limN,T→∞−N2T−1∂2ELNT(θ0,a^(θ0))∂θ∂θ⊤,

we are in a position to show

**Theorem** **6.** *Under Assumptions 1 and 2,*

(14) $$\frac{\sqrt{NT}\left(\hat{\theta }-\theta^{0}\right)-J_{0}^{-1}\left(\theta \right)}{J_{0}{\left(\theta \right)}^{1/2}}\overset{D}{\to }N\left(0,I_{\dim(\theta )}\right).$$

To converge to a normal distribution, the difference between estimator $\hat{\theta }$ and true value $\theta^{0}$ has to be bias-corrected and rated proportionally to the number of nodes *N* and time *T*. In the dense network setting considered here, $\theta^{0}$ is estimated based on the observed linking decisions about N(N−1) potential links. Therefore, the rate of convergence $\sqrt{NT}$ is the conventional parametric rate corresponding to the sample size [5,7].

We finalize this section showing some functional dimensions of the entropy function, given by

**Theorem** **7.** *Under Assumptions 1 and 2, we have that:*

(*i*)
$$H\left(C\right)\leq \frac{\rho_{kl,N}}{K}\log\left(\frac{K(N-2)}{\rho_{kl,N}}\right)-\sum_{i=1}^{N-1}\frac{l_{i(i+1),t}^{\theta ,a_{N}}}{K}\log\left(\frac{l_{i(i+1),t}^{\theta ,a_{N}}}{K}\right)-\sum_{i=1}^{N-1}\frac{l_{iN,t}}{K}\log\left(\frac{l_{iN,t}^{\theta ,a_{N}}}{K}\right),$$
  *where*
$$\rho_{kl,N}=\sum_{k=1}^{N-1}\sum_{l=k+1}^{N}l_{kl,t}-\sum_{k=1}^{N-1}\left(l_{k(k+1),t}^{\theta ,a_{N}}+l_{kN,t}^{\theta ,a_{N}}\right)$$
  *and $K=\sum_{i<j}\sum_{t=1}^{T}\log\left(\frac{z_{ij,t}\left(\theta ,a_{N}\right)}{{\left(1+z_{ij,t}\left(\theta ,a_{N}\right)\right)}^{2}}\right).$*
(*ii*) *If $F=\{c_{ij,t^{\prime }}:t^{\prime }<t\}$ and $F^{\prime }=\left\{c_{ij,t^{\prime }}^{\prime }:t^{\prime }<t\right\}$ are two filtrations with respect to A, then*
(15) $$H\left(C\right)\geq -\sum_{i<j}\varrho_{ij,F}\log\left(\varrho_{ij,F^{\prime }}\right)-\frac{1}{\ln(2)}\sum_{i<j}\frac{{\left(\varrho_{ij,F}\right)}^{2}-\varrho_{ij,F}\varrho_{ij,F^{\prime }}}{\varrho_{ij,F^{\prime }}},$$
  *where $\varrho_{ij,F}=\frac{l_{ij,F}^{\theta ,a_{N}}}{\sum_{i<j}l_{ij,F}^{\theta ,a_{N}}}$ and $\varrho_{ij,F^{\prime }}=\frac{l_{ij,F^{\prime }}^{\theta ,a_{N}}}{\sum_{j<i}l_{ij,F^{\prime }}^{\theta ,a_{N}}}.$*

Theorem 7 states that the entropy H(C) of the dynamic network *C* is bounded by the mutual information between successive states of the filtrations F and $F^{\prime }$. This means that as the states of the network become more predictable and related to each other, the entropy decreases, implying greater structure and order in the network. Conversely, if the states are more independent and random, the entropy increases, reflecting a more chaotic and less predictable structure in the network.

## 4. Benchmark and Simulations

In this section, we studied the finite sample performance of procedures in Monte Carlo simulations, where the programming language used for these simulations is Matlab. We compared the development and robustness of our network formation model using the Erdös–Rényi [18] and Barabási–Albert [19]-type networks. The Barabási–Albert network was generated with a connection probability of 0.5 and a new number of links in each period equal to five. These comparisons were made with the metrics of degree distribution, clustering coefficient, and entropy value. The experiment was based on the latent index formation rule with specification

$$C_{ij,t}=1\left\{\sum_{p=1}^{10}\alpha_{p}C_{ij,t-p}+X_{it}X_{jt}^{⊤}\beta +A_{ij}-\epsilon_{ij,t}\geq 0\right\}$$

Here, $\beta^{0}=0.5$, $\alpha^{0}$ is a random vector with a norm of less than one and $X_{i}\in \left\{-1,1\right\}$, i=1,…,N being independent and identically distributed random variables simulated by $X_{i}=1-2\cdot 1\left\{i\;\;\mathrm{is}\;\mathrm{even}\right\}$, with size networks of 100, 150, 200, 250 and 500. For larger sample sizes, the behavior of the entropy function is, on average, similar. With this specification, nodes with an even index prefer links to nodes with an even index over links to nodes with an odd index, and vice versa for nodes with an odd index. Through 1000 repetitions of the experiment, we show the reproducibility and dynamics of the constructed networks. A 15-step time experiment was proposed. In addition, $A_{ij}=\left|A_{i}-A_{j}\right|$ with $A_{i}=\left(\frac{N-i}{N-1}\right)\log N$ for all i=1,…,N. The descriptive characteristics of the network formation are shown in Table 1. Based on Table 1, we can perform a comparative analysis between the three generated networks.

1. Mean Degree: The mean degree represents the average number of connections that nodes have in the network. In the simulated network, the mean degree decreases as the network size increases, suggesting that nodes tend to be less connected to each other. This could be influenced by the parameters of the network generation model, such as α, β, and *p*, which affect the probability of forming new connections at each time step. On the other hand, the Erdös–Rényi and Barabási–Albert networks maintain their mean degree relatively constant, indicating that their connection generation process is not strongly influenced by network size.
2. Standard Deviation of Degree: The standard deviation of the degree measures the variability in the number of connections that nodes have in the network. In the simulated network, the standard deviation of the degree tends to decrease as the size of the network increases, implying that node degrees become more homogeneous. This could be a desirable feature in some contexts, as it indicates that the simulated network tends to have a more uniform degree distribution, which is associated with greater robustness and stability in its structure.
3. Clustering Coefficient: The clustering coefficient measures the proportion of connections that exist between the neighbors of a given node. In the simulated network and Erdöss–Rényi networks, the clustering coefficient tends to decrease as the size of the network increases. This suggests that nodes tend to be less interconnected compared to smaller networks. On the other hand, in the Barabási–Albert network, the clustering coefficient remains at one, indicating that neighboring nodes are highly connected. This result is characteristic of Barabási–Albert scale-free networks, where new nodes tend to preferentially connect to existing nodes with higher degrees, resulting in high clustering among the neighbors of each node.

Regarding the convergence order, it is observed that the simulated network exhibits an intermediate behavior between Erdös–Rényi and Barabási–Albert networks in terms of mean degree and clustering coefficient. While Erdös–Rényi networks are more homogeneous and less clustered, and Barabási–Albert networks are more heterogeneous and highly clustered, the simulated network shows intermediate characteristics, making it suitable for representing systems that contain elements of both tendencies.

Concerning entropy, we validated the development of entropy H(C) across the same number of network sizes over three time periods. We compared the results with Erdös-Rényi entropy [57] and Shannon entropy [58]. Table 2 summarizes the results obtained from 1000 simulations. The analysis shows that entropy H(C) performs consistently well across various network sizes and time periods. It demonstrates competitive values compared to Shannon entropy and outperforms Erdös–Renyi entropy significantly. The results indicate that H(C) is a reliable and effective measure to capture the information flow in network dynamics. The lower values obtained by H(C) compared to Shannon entropy suggest that it provides a more informative representation of the network’s complexity. Furthermore, the increasing trend of H(C) with network size indicates that it effectively captures the growing complexity of larger networks, indicating that larger networks tend to have more structure and order. Overall, these findings support the usefulness of H(C) as an entropy measure for analyzing network dynamics and information flow. Higher Shannon entropy values indicate greater diversity or complexity within the networks. In this context, Shannon entropy decreases as the size of the network increases, which implies greater self-organization and less uncertainty within larger networks.

## 5. Empirical Application

In this section, we apply our dynamic network formation model (Equation (1)) to the 18S rRNA gene amplicon dataset from a study by Hu et al. [40]. This application has focused the microbial network identification. Seawater samples were collected from a depth of 15 m every 4 h following a Lagrangian sampling schematic in an anticyclonic eddy in the North Pacific Subtropical Gyre, as a part of the Simons Collaboration on Ocean Processes and Ecology (SCOPE, http://scope.soest.hawaii.edu/) cruise efforts in July 2015. Some species with taxonomical classification of RNA OTUs are shown in Table 3.

### 5.1. Analytical Processes

We examined the influence of species richness, specifically focusing on the relative rather than absolute frequency of OTUs. This simplicity forms the primary homophilic structure governing interactions among species taxa in this microbial context, where species engage based on their relative abundances. Subsequently, we applied the Community Louvain algorithm to identify the microbial communities participating in various interactions during each sampling period. To validate the algorithm’s findings, we conducted null modularity calculations with 1000 replicates to assess the statistical significance and distinctiveness of the identified communities within the networks. Additionally, we considered community uniformity and similarity across the sampling periods. To confirm sample dissimilarity, we conducted multiple ANOVA tests and employed the Jaccard test. Our analysis encompassed sensitivity, interaction intensity, and the effect of parameters observable and non-observable on microbial diversity. Computational cost allowed us evaluation of six samples. Samples were collected using 10 L Niskin bottles mounted on a CTD rosette at 6 a.m., 10 a.m., 2 p.m., 6 p.m., 10 p.m., and 2 a.m. Corresponding temperature, salinity, dissolved oxygen, and chlorophyll *a* data were derived from the same CTD casts. The input data are presented in the form of sequential count tables, where each column represents a sample, and each row represents a taxonomic designation (OTU or transcription ID) with sequence count or read coverage abundance per taxon. Global singletons (where a single OTU appears with a frequency of 1 in the entire dataset sequence) are removed. Out of a total of 3831 Taxa observed, 1779 are eliminated.

### 5.2. Results

Incorporating the details outlined above, along with the dynamic network formation model (1), we present the following results.

#### 5.2.1. Calculating Sensitivity and Specificity, Effect of Interaction Intensity

The interaction network and co-occurrence network were compared to each other to determine the sensitivity and specificity of the constructed co-occurrence network in detecting direct (first-order) interactions [25]. For this calculation, a true positive (TP) was indicated by the presence of an edge in the co-occurrence network that had the same sign as in the interaction network (when using association metrics with sign). A false positive (FP) represented an edge in the co-occurrence network that was not present in the interaction network. A false negative (FN) denoted an edge in the interaction network that was absent in the co-occurrence network. A true negative (TN) was the absence of an edge in both the interaction and co-occurrence networks. Sensitivity was defined as TP/(TP + FN), and specificity was defined as TN/(TN + FP). In cases where two species interacted with each other with different signs, the interaction with the larger absolute value was considered to be the sign of the net interaction. In addition, we calculated each precision as TP/(TP + FP), and F1 score as 2×(precision×sensibility)/(precision+sensibility).

The similarity of species had a large effect on network sensitivity (see Table 4). Though specificity remained high at similarities ranging from 89% to 90%, the sensitivity increased through this range within creasing similarity. Samples with relatively high similarity in species membership were therefore useful for constructing sensitive networks. Many real microbial communities have a lower percentage of shared taxa, but this is largely due to under sampling of rare species [59]. The F1-score, which reflects the balance between precision and recall in measuring species interaction or co-occurrence, consistently indicates strong performance throughout the day. An F1-score of 0.637 indicates a good balance between precision and recall for species interaction or co-occurrence at 6 a.m. This means that the model or method used to measure species interaction performs well in identifying both positive (species interactions) and negative (absence of interactions) cases at this time. At other time points, including 10 a.m., 2 p.m., 6 p.m., 10 p.m., and 2 a.m., the F1-scores range from 0.635 to 0.644. These values suggest that the employed method effectively identifies species interactions, with a particularly noteworthy performance during the nighttime hours at 2 a.m. Overall, the F1-score results highlight the method’s robustness in assessing species interactions across different times of the day.

#### 5.2.2. Effect of Interaction Intensity in the Communities

Once the co-occurrence networks between species are constructed, we investigate the community structure that these interactions generate. In each sampling instance, we identify microbial communities based on the interaction of the corresponding species. These interaction networks of communities evolve with each sampling, both in terms of the number of communities and the composition of these communities. The depth of this identification is carried out at seven taxonomic levels. The original dataset comprises eight taxonomic levels, as described in Table 3. The sampling time reveals preferences in the interactions among certain communities. For instance, some of the microbial communities tend to be more inclined to interact during the day, likely due to the increased presence of the 18S rRNA gene within their taxonomy. Tukey–Kramer tests were conducted in this sampling. All tests resulted in p<0.001 in favor of rejecting the null hypothesis: there is no statistically significant evidence in the mean of the compared communities. The randomness test is performed on the degree distribution at all sampling points. In all of these, we find a *p*-value <0.001, indicating that the biological network formation structure does not follow a random structure. The modularity test based on 1000 permutations yields a *p*-value <0.001. This indicates that the formation of these communities is robust and the interactions are strongly cohesive at each sampling.

**Figure 2:**
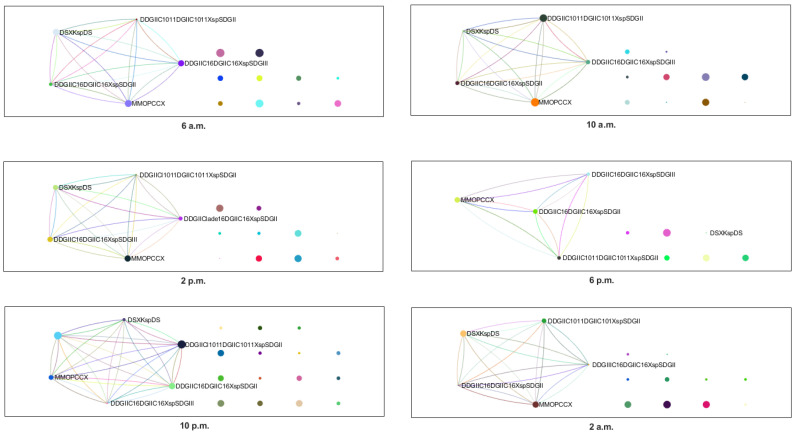
Identifying interaction in communities in sampling networks.

Figure 2 shows the different interaction networks of microbial communities. The relative frequency of the communities is described by the size of their respective node. In this work, notation DSDGIIC16DGIIC16DGIIC16XspSDGII refers to the microbial community *Dinophyta-Syndiniales-Dino-Group-II-Clade-16-Dino-Group-II-Clade16Xsp.-Syndiniales-Dino-Group-II*, nomenclature DSDGIIC16DGIIIC16XspSDGII refers to *Dinophyta-Syndiniales-Dino-Group-II-Clade-16-Dino-Group-III-Clade16Xsp-Syndiniales-Dino-Group-II*, nomenclature DSDGIIC1011DGIIC1011XspSDGII refers to *Dinophyta-Syndiniales-Dino-Group-II -Clade-10-11-Dino-Group-II-Clade-10-11Xsp-Syndiniales-Dino-Group-II*, MCMOPCCX to *Metazoa-Craniata-Mammalia-Ochotona-princeps-Craniata-CraniataX*, DDSDKspDS to *Dinophyta-Dinophyceae-SuessialesX-Karlodiniumsp-Dinophyceae-Suessiales* and DDSXSspDS to *Dinophyta-Dinophyceae-SuessialesX-Symbiodiniumsp-Dinophyceae-Suessiales*. Unlabeled nodes refer to unidentified communities according to the seven sequencing depth levels.

#### 5.2.3. Effects of Parameters on Microbial Diversity

Microbial communities in different environments can vary widely in their composition and structure. Though the experimenter cannot necessarily influence ecological parameters, it is valuable to know which factors may cause problems in co-occurrence network inference. We considered the effect of species richness, community evenness, and similarity of communities across sampling sites.

Our analysis suggests that community evenness does not directly affect co-occurrence network sensitivity and specificity. However, it may have an indirect effect because uneven communities require increased sampling depth in order to detect the real species richness, and if this is inadequate, then the number of detected species (i.e., the effective richness) is reduced. The diversity of communities between different sites can be calculated via a variety of metrics [60]. We used a simple and intuitive metric to quantify the similarity of communities at different sampling sites: the average percentage of species shared between any two sites (i.e., the Jaccard similarity). The similarity of communities had a large effect on network sensitivity. The Jaccard index for all community networks is 0.017, indicating a dynamic configuration in the networks and thus in the microbial structure. This is of utmost importance due to the intrinsic biological complexity of genomic structure, considering that some of the taxonomic properties of the 16S rRNA gene are more expressive at certain times of the day.

#### 5.2.4. Effect of the Non-Observable

The communities evaluated so far have not been in a steady state, representative of many complex communities [61,62]. Therefore, we investigated the ways in which the variability in unobservable site properties influences the inference of each network of communities. To achieve this, we introduced random variations in the carrying capacity of each species at each site, which can be interpreted as an introduction of between-site heterogeneity. This addition of inter-site heterogeneity, where each species has varying advantages, introduced “noise” to the dataset. Nevertheless, we mitigated the impact of the unobservable factors using Theorem 4.

Table 5 shows the variation of network statistics as the level of heterogeneity changes. We observed that the number of microbial communities varies depending on the time of day and the formula for unobserved heterogeneity used. At 6 a.m. and 10 p.m., the number of communities was lower when formula $\frac{N-i}{N-1}\log\left(\log\left(N\right)\right)$ was applied, which could indicate a higher cohesion among communities at those hours. In contrast, at 2 p.m., regardless of the formula, a constant number of communities was maintained, suggesting a more robust structure. Regarding the average node degree in the networks, there was no clear pattern of increase or decrease based on the time of day or the formulation of unobserved heterogeneity. The values fluctuated under all conditions, implying natural variability in microbial interactions. Finally, the density of the networks showed significant variations. For example, at 10 a.m. and 2 p.m., the density was relatively low, implying a lower proportion of possible connections in these networks. In contrast, at 6 p.m., a higher density was observed, suggesting greater interconnection among microbial species at that time.

## 6. Discussion and Conclusions

Motivated to explore the field of CNA, we study the introduction of a latent interaction index, addressing the limitations inherent in traditional compositional similarity indices, taking into account both observed and unobserved heterogeneity per node, particularly in the context of large and complex networks.

This index addresses a limitation in network formation, namely interdependence. The study of complex network formation in the presence of interdependencies is one of the focal points of recent theoretical and empirical research on networks [5,7,16]. However, with the exception of Graham’s [7] and Dzemski’s [5] models, none of these papers incorporate unobserved correlated heterogeneity within the modeling framework, unlike the approach used here. The results obtained through the development of this index (Theorems 1 and 2) demonstrate uniform consistency with respect to the homophily parameter vector and fixed effects. This assures us that the proposed index yields statistically replicable results, in line with the principles of the law of large numbers and its applicability across various domains [2,25].

Together with the above, we formulate a Shannon-type entropy measure to quantify network density. We further establish optimal boundaries for this measurement by utilizing insights from network topology. Additionally, we present asymptotic properties of pointwise estimation using this entropy function. This analytical approach allows us the application of scrutiny on the structural dynamics of composition, offering valuable insights into the intricate interactions within the network. Here, it is important to note the relevance of dyads contributing to this measure, as a consequence of both observed and unobserved factors. In contrast to some studied entropy measures that do not take these characteristics into account [23,27,29,34,36], it might be more useful and comprehensive for future research in various fields to conduct a deeper exploration of what other factors and dimensions could potentially influence the contributions of dyads in the network and, consequently, network entropy.

The results indicate that as network states become more predictable and interconnected, network entropy decreases. This decrease in entropy signifies a greater degree of structure and order within the network. Conversely, when network states exhibit greater independence and randomness, entropy increases, reflecting a more chaotic and less predictable network structure. These findings align with previous research on the interplay between network structure and entropy [13,14,63].

The application of the Shannon-type entropy function provides a robust measure for quantifying network complexity. By establishing optimal bounds for entropy estimation based on network topology, we ensure the accuracy of our analysis and enhance our ability to distinguish networks with varying complexity levels. This contributes to a more nuanced understanding of network dynamics and interactions. Simulations and comparisons with Erdös–Rényi and Barabási–Albert-type networks, in addition to the utilization of Erdös–Rényi and Shannon-type entropy, further validate the effectiveness of our proposed method. Our results demonstrate that the proposed index successfully distinguishes between networks with different degrees of complexity, even outperforming classical models in certain cases [18,19].

Despite the inherent complexity of microbiological data [40,61], our method offers a promising avenue for studying and comprehending the intricate relationships within these interaction networks and their implications under various parameter specifications. The ecological results presented here are currently under discussion with experts in the field. However, we acknowledge the possibility of simplifications and extensions of the model proposed here.

The theoretical results presented in this article allow us formulation of two statements. First, the interdependence structure in forming complex networks should not be independent of the objective parameters and unobservable node effects. This would enable researchers to discover causal relationships based on these parameters and the network formation itself, complementing some of the discussed network models [37,38]. Second, entropy measures on network structures could be more robust and consistent if only the dyads influencing their structure were considered. It is well-known that biased estimates in entropy measures of networks arise from the influence of *false dyads* on the system [64]. The entropy metric presented here is based solely on the contributing dyads of the network.

In conclusion, this approach enables us to capture both observed and unobserved heterogeneity per node, providing a more comprehensive understanding of interactions within ecological communities and other intricate networks. The proposed latent interaction index proves to be an invaluable tool for characterizing the structural dynamics of networks. Additionally, it is feasible to design a test to evaluate interdependencies in link formation. It is more plausible to assume that these interdependencies establish a bounded degree between pairwise interactions [21,24]. The proposed model provides feasibility and evidence of how to incorporate these interdependencies, which in many cases are probabilities conditioned on triads (groups of three nodes) [5,7]. It is worth noting that these probabilities introduce a bias in the linkage decision [6]. While work has been extensive in reducing this bias in mono-nodal estimation [16,46,47], little is known about multi-nodal structures. This inherent uncertainty led to the introduction of the entropy function studied here. It possesses the property of reflecting parameter estimates as a function of the true parameters, meaning that the estimated entropy converges to the true entropy. This finding could be a valuable contribution to the challenge of multinodal estimates.

## Figures and Tables

**Table 1:** Network statistics.

Network	Size Network	Mean Degree	Standard Deviation	Cluster Coeficient	Standard Deviation (Cluster Coefficient)
Simulate	100	0.50	3.01	0.91	0.15
Erdös–Rényi	100	0.10	1.40	0.11	0.01
Barabási–Albert	100	0.28	0.25	1.01	0.24
Simulate	150	0.33	2.19	0.97	0.18
Erdös–Rényi	150	0.05	1.78	0.11	0.02
Barabási–Albert	150	0.10	0.15	1.02	0.25
Simulate	200	0.27	1.73	0.92	0.19
Erdös–Rényi	200	0.06	0.91	0.08	0.01
Barabási–Albert	200	0.12	0.08	0.56	0.14
Simulate	250	0.22	1.44	0.94	0.16
Erdös–Rényi	250	0.04	0.44	0.08	0.05
Barabási–Albert	250	0.20	0.06	0.01	0.16
Simulate	500	0.20	0.82	0.98	0.14
Erdös–Rényi	500	0.09	0.44	0.09	0.008
Barabási–Albert	500	0.13	0.01	0.01	0.13

**Table 2:** Network entropy values.

Network	Size Network			
	**100**	**200**	**250**	**500**
H(C)	4.27	4.36	4.05	3.33
Shannon Entropy	10.11	11.50	11.94	14.34
Erdös-Rényi Entropy	401.3	523.1	559.4	689.9

**Table 3:** Taxonomic identities.

Taxonomic Group		Taxonomic Detail of RNA OTUs
Alveolates	Ciliates	*Phyllopharyngea*, *Spirotrichea*, *Litostomatea, Prostomatea*, *Oligohymenophorea*, and *Colpodea*
	Dinoflagellates	*Symbiodinium*, *Gyrodinium*, *Protoperidinium, Prorocentrum*, *Dinophysis*, *Gymnodinium, Heterocapsa*, *Apicoporus*, *Suessiales*, *Azadinium, Blastodinium*, *Chytriodinium*, *Peridinium, Amphisolenia*, *Phalacroma*, *Amphidinium*, and *unclassified Dinophyceae.*
	Syndiniales	*Dino-Group-I*, *Dino-Group-II*, *Dino-Group-III*, and *Dino-Group-V*
Archaeplastids	Chlorophytes	*Chlorodendrophyceae*, *Pyramimonadales*, and *Prasino-Clade-VII*
	Other	*Heliconia*.
Rhizaria	Acantharia	*Hexaconus*, *Chaunacanthida*, *Acantharea*, *Amphilonche, Staurolithium*, *Acanthocolla*, and *Heteracon.*
	Cercozoa	*Protaspa*.
Opisthokont	Fungi	Other-unclassified
	Metazoa	*Arthropoda*, *Mollusca*, *Annelida*, and *Urochordata*.

**Table 4:** Metric values of the sample of the network.

Network Time	Metric						
	**Entropy**	**Transitivity**	**Mean Degree**	**Sensitivity**	**Specifity**	**Precision**	**F1-Scores**
6 a.m.	$1.19\times 10^{8}$	0.172	49.4	0.905	0.098	0.493	0.637
10 a.m.	$1.23\times 10^{8}$	0.171	50.1	0.895	0.101	0.503	0.644
2 p.m.	$1.22\times 10^{8}$	0.176	40.5	0.896	0.096	0.501	0.643
6 p.m.	$1.21\times 10^{8}$	0.173	25.5	0.900	0.100	0.490	0.635
10 p.m.	$1.18\times 10^{8}$	0.167	24.6	0.891	0.103	0.494	0.636
2 a.m.	$1.24\times 10^{8}$	0.173	16.8	0.906	0.102	0.496	0.641

**Table 5:** Effect of the non-observable on microbial communities.

Network Time		$A_{i}$		
		$\frac{N-i}{N-1}\log\left(\log\left(N\right)\right)$	$\frac{N-i}{N-1}\log N^{1/2}$	$\frac{N-i}{N-1}\log N$
6 a.m.	Communities	21	15	15
	Mean degree	2.86	2.67	2.67
	Denstity	0.14	0.19	0.19
10 a.m.	Communities	28	15	21
	Mean degree	3	2.67	2.86
	Denstity	0.11	0.19	0.14
2 p.m.	Communities	21	21	21
	Mean degree	2.86	2.86	2.86
	Denstity	0.14	0.14	0.14
6 p.m.	Communities	15	21	15
	Mean degree	2.67	2.86	2.67
	Denstity	0.19	0.14	0.19
10 p.m.	Communities	15	15	15
	Mean degree	2.67	2.67	2.67
	Denstity	0.19	0.19	0.19
2 a.m.	Communities	28	21	15
	Mean degree	3	2.86	2.67
	Denstity	0.11	0.14	0.19

## Data Availability

No new data were created or analyzed in this study. Data sharing is not applicable to this article.

## Appendix A

### Appendix A.1. Proofs

To ensure the comprehensiveness of the present work, this part is reserved for the proofs for Lemma 1 and Theorems 1–7.

**Proof** **of** **Theorem** **1.** From Hoeffding’s inequality, ∀ϵ>0,

$$\mathrm{Pr}\left(\left|\sum_{i<j}\sum_{t=1}^{T}\left(C_{ij,t}-p_{ij,t}\right)\geq \epsilon \right|\right)\leq 2\exp\left(\frac{-2\epsilon^{2}}{{\left(1-2\kappa \right)}^{2}}\right),$$

where κ∈(0,1) such that $p_{ij,t}\left(\theta ,a_{N}\right)\in \left(\kappa ,1-\kappa \right)$. Setting $\epsilon =\sqrt{\ln(NT)}$, we have

$$\mathrm{Pr}\left(\left|\sum_{i<j}\sum_{t=1}^{T}\left(C_{ij,t}-p_{ij,t}\right)\geq \sqrt{\ln(NT)}\right|\right)\leq 2\exp\left(\frac{-2\ln(NT)}{{\left(1-2\kappa \right)}^{2}}\right).$$

Here, $2\exp\left(\frac{\ln\left(\frac{1}{{\left(NT\right)}^{2}}\right)}{{\left(1-2\kappa \right)}^{2}}\right)=\frac{2}{{\left(NT\right)}^{2}}\exp\left(\frac{1}{{\left(1-2\kappa \right)}^{2}}\right)=O\left({\left(NT\right)}^{-2}\right)$. Therefore,

$$\underset{1\leq i,j\leq N}{\sup}\underset{1\leq t\leq T}{\sup}\left|\sum_{i<j}\sum_{t=1}^{T}\left(C_{ij,t}-p_{ij,t}\right)\leq \epsilon \right|$$

with probability $1-O\left({\left(NT\right)}^{-2}\right)$. □

**Proof** **of** **Theorem** **2.** We note that

E∑i<jlij,tθ,aN|F=N2−1T∑i<j∑t=1Tcij,tlog(zij,t(θ,aN))−log(1+zij,t(θ,aN))|F.

The last equation can be written as

E∑i<jlij,tθ,aN|F=N2−1T∑i<j∑t=1Tlogzij,t(θ,aN)1+zij,t(θ,aN).

Via Theorem 4.2.2 from [48], we have

$$\frac{1}{T}\sum_{t=1}^{T}\log\left(\frac{z_{ij,t}\left(\theta ,a_{N}\right)}{1+z_{ij,t}\left(\theta ,a_{N}\right)}\right)\overset{\mathrm{Pr}}{\to }E\left[\log\left(\frac{z_{ij,t}\left(\theta^{0},a_{N}^{0}\right)}{1+z_{ij,t}\left(\theta^{0},a_{N}^{0}\right)}\right)\right],$$

uniformly in θ∈Θ when $E\left(\sup_{\theta \in \Theta }|\log\left(\frac{z_{ij,t}\left(\theta ,a_{N}\right)}{1+z_{ij,t}\left(\theta ,a_{N}\right)}\right)|\right)$ is finite. Then,

E∑i<jlij,tθ,aN|F→PrN2−1∑i<jElogzij,t(θ0,aN0)1+zij,t(θ0,aN0),

uniformly in θ∈Θ. □

**Proof** **of** **Theorem** **3.** Since $\hat{\theta }$ is based on contribution $l_{ij,t}^{\hat{\theta },a_{N}}$, which, by Theorem 2, converges in probability to a monotonic transformation of vector $\theta^{0}$, from Theorem 4.2.1 in [48], this implies that $\lim_{N,T\to +\infty }\hat{\theta }=\theta^{0}$ and, therefore, the variance of $∥\hat{\theta }-\theta^{0}∥$ proceeds to zero as N,T approaches positive infinity. We let δ>0 be a fixed small constant. Then, via Chebyshev’s inequality, we have

$$\lim_{N,T\to +\infty }\mathrm{Pr}(∥\hat{\theta }-\theta^{0}∥\geq \delta )\leq \lim_{N,T\to +\infty }\frac{\mathrm{Var}(∥\hat{\theta }-\theta^{0}∥)}{\delta^{2}}=0.$$

This means that the probability of $\hat{\theta }$ being within a small neighborhood of $\theta^{0}$ approaches 1 as N,T becomes large. To determine the rate of convergence, we can express this difference as $∥\hat{\theta }-\theta^{0}∥=O\left({\left(NT\right)}^{-1/2}\right)$. This indicates that the convergence rate is at least $O\left({\left(NT\right)}^{-1/2}\right)$. □

**Proof** **of** **Lemma** **1.** Let us calculate difference

$$\begin{array}{cc} {\hat{\delta }}_{ij}\left(x\right)-\delta_{ij}\left(x\right) & =\frac{1}{NT}\sum_{l=1}^{N}\sum_{t=1}^{T}\left(\left(C_{il,t}-C_{jl,t}\right)\tilde{K}\left(\frac{x-X_{l}}{h(N)}\right)-E\left(C_{il,t}-C_{jl,t}|X,A,D\right)\right)\\ & =\frac{1}{NT}\sum_{l=1}^{N}\sum_{t=1}^{N}(C_{il,t}\tilde{K}\left(\frac{x-X_{l}}{h(N)}\right)-E\left(C_{il,t}|X,A,D\right)-\\ & -(C_{jl,t}\tilde{K}\left(\frac{x-X_{l}}{h(N)}\right)-E\left(C_{il,t}-C_{jl,t}|X,A,D\right)) \end{array}$$

by applying Hoeffding’s inequality twice:

$$\begin{array}{cc} \left|{\hat{\delta }}_{ij}\left(x\right)-\delta_{ij}\left(x\right)\right| & \leq \frac{1}{NT}(\left|\sum_{l=1}^{N}\sum_{t=1}^{T}C_{il,t}\tilde{K}\left(\frac{x-X_{l}}{h(N)}\right)-E\left(C_{il,t}|X,A\right)\right|+\\ \; & +\left|\sum_{l=1}^{N}\sum_{t=1}^{T}C_{jl,t}\tilde{K}\left(\frac{x-X_{l}}{h(N)}\right)-E\left(C_{il,t}-C_{jl,t}|X,A\right)\right|)=O_{p}\left({\left(NT\right)}^{-1}\right). \end{array}$$

□

**Proof** **of** **Theorem** **4.** We denote ${\tilde{L}}_{NT}\left({\hat{\delta }}_{ij}\left(x_{l}\right)\right)=\frac{\ell_{NT}\left({\hat{\delta }}_{ij}\left(x_{l}\right)\right)}{\sum_{i<j}\ell_{NT}\left({\hat{\delta }}_{ij}\left(x_{l}\right)\right)}$ with $\ell_{NT}\left(x\right)=L\left(\frac{x}{\sigma_{N}}\right)$.
First, we note that

$$\begin{array}{cc} {\tilde{L}}_{NT}\left({\hat{\delta }}_{ij}\left(x_{l}\right)\right)X_{il,t}X_{jl,t}^{⊤}\hat{\theta } & ={\tilde{L}}_{NT}\left({\hat{\delta }}_{ij}\left(x_{l}\right)\right)\theta +X_{il,t}X_{jl,t}^{⊤}\left(\hat{\theta }-\theta \right){\tilde{L}}_{NT}\left({\hat{\delta }}_{ij}\left(x_{l}\right)\right)+\\ \; & +X_{il,t}X_{jl,t}^{⊤}\theta \left({\tilde{L}}_{NT}\left({\hat{\delta }}_{ij}\left(x_{l}\right)\right)-{\tilde{L}}_{NT}\left(\delta_{ij}\left(x_{l}\right)\right)\right). \end{array}$$

Since $X_{il,t}$, $X_{jl,t}$, and $\delta_{ij}\left(x_{l}\right)$ are bounded for all l=1,…,N, by mean value expansion of $\ell_{NT}\left({\hat{\delta }}_{ij}\left(x_{l}\right)\right)$ around $\ell_{NT}\left(\delta_{ij}\left(x_{l}\right)\right)$, we have $\ell_{NT}\left({\hat{\delta }}_{ij}\left(x_{l}\right)\right)-\ell_{NT}\left(\delta_{ij}\left(x_{l}\right)\right)=\ell_{NT}^{\prime }\left(\xi \right)\left|{\hat{\delta }}_{ij}\left(x_{l}\right)-\delta_{ij}\left(x_{l}\right)\right|$. Here, $\left|\ell_{NT}\left({\hat{\delta }}_{ij}\left(x_{l}\right)\right)-\ell_{NT}\left(\delta_{ij}\left(x_{l}\right)\right)\right|=O_{p}(\sup\left|{\hat{\delta }}_{ij}\left(x_{l}\right)-\delta_{ij}\left(x_{l}\right)\right|)$. By Lemma 1, $\left|\ell_{NT}\left({\hat{\delta }}_{ij}\left(x_{l}\right)\right)-\ell_{NT}\left(\delta_{ij}\left(x_{l}\right)\right)\right|=O_{p}\left({\left(NT\right)}^{-1}\right)$. By Theorem 1 and using of Chevyshev inequality,

$$\mathrm{Pr}\left(|X_{il,t}X_{jl,t}^{⊤}\left(\hat{\theta }-\theta \right){\tilde{L}}_{nt}\left({\hat{\delta }}_{ij}\left(x_{l}\right)\right)|>\epsilon \right)\leq E\left(\frac{1}{\epsilon^{2}}{\left(X_{il,t}X_{jl,t}^{⊤}\left(\hat{\theta }-\theta \right){\tilde{L}}_{nt}\left({\hat{\delta }}_{ij}\left(x_{l}\right)\right)\right)}^{2}\right),$$

for all $\epsilon >0,\forall \theta ,\hat{\theta }\in \Theta .$ Now, from the Cauchy–Schwartz inequality,

(A1) $$\begin{array}{cc} E{\left(X_{il,t}X_{jl,t}^{⊤}\left(\hat{\theta }-\theta \right){\tilde{L}}_{nt}\left({\hat{\delta }}_{ij}\left(x_{l}\right)\right)\right)}^{2} & \leq E{\left(X_{il,t}X_{jl,t}^{⊤}\left(\hat{\theta }-\theta \right){\tilde{L}}_{nt}\left({\hat{\delta }}_{ij}\left(x_{l}\right)\right)\right)}^{2}\cdot \\ \; & \cdot E{\left(X_{ik,t}X_{jk,t}^{⊤}\left(\hat{\theta }-\theta \right){\tilde{L}}_{nt}\left({\hat{\delta }}_{ij}\left(x_{k}\right)\right)\right)}^{2}. \end{array}$$

In addition, $E{\left(X_{ik,t}X_{jk,t}^{⊤}\left(\hat{\theta }-\theta \right){\tilde{L}}_{nt}\left({\hat{\delta }}_{ij}\left(x_{k}\right)\right)\right)}^{2}=O_{p}\left(\sigma_{n}{\left(NT\right)}^{-1}\right)$. Second, we note that

$$\begin{array}{cc} \sum_{i<j}\sum_{t}X_{il,t}X_{jl,t}^{⊤}\theta \left({\tilde{L}}_{nt}\left({\hat{\delta }}_{ij}\left(x_{l}\right)\right)-{\tilde{L}}_{nt}\left(\delta_{ij}\left(x_{l}\right)\right)\right)= & \sum_{i<j}\sum_{t}X_{il,t}X_{jl,t}^{⊤}\theta \times \\ & \times \left[\frac{\ell_{NT}\left({\hat{\delta }}_{ij}\left(x_{l}\right)\right)}{\sum_{i<j}\ell_{NT}\left({\hat{\delta }}_{ij}\left(x_{l}\right)\right)}-\frac{\ell_{NT}\left(\delta_{ij}\left(x_{l}\right)\right)}{\sum_{i<j}\ell_{NT}\left(\delta_{ij}\left(x_{l}\right)\right)}\right]\\ & \leq \frac{\sum_{i<j}\sum_{t}\ell_{NT}\left({\hat{\delta }}_{ij}\left(x_{l}\right)\right)X_{il,t}X_{jl,t}^{⊤}\theta }{\sum_{i<j}\ell_{NT}\left({\hat{\delta }}_{ij}\left(x_{l}\right)\right)}\cdot \\ & \cdot \frac{\sum_{i<j}\sum_{t}\ell_{NT}\left(\delta_{ij}\left(x_{l}\right)\right)-\ell_{NT}\left({\hat{\delta }}_{ij}\left(x_{l}\right)\right)}{\sum_{i<j}\ell_{NT}\left(\delta_{ij}\left(x_{l}\right)\right)}+\\ & +\frac{\sum_{i<j}\sum_{t}\ell_{NT}\left(\delta_{ij}\left(x_{l}\right)\right)-\ell_{NT}\left({\hat{\delta }}_{ij}\left(x_{l}\right)\right)X_{il,t}X_{jl,t}^{⊤}\theta }{\sum_{l}\ell_{NT}\left(\delta_{ij}\left(x_{l}\right)\right)}\\ & =O_{p}\left({\left(NT\right)}^{-1}\right)+\\ & +\frac{\sum_{i<j}\sum_{t}\left(\ell_{NT}\left(\delta_{ij}\left(x_{l}\right)\right)-\ell_{NT}\left({\hat{\delta }}_{ij}\left(x_{l}\right)\right)\right)\bar{X(\theta )}}{\sum_{i<j}\ell_{NT}\left(\delta_{ij}\left(x_{l}\right)\right)} \end{array}$$

with $\sup_{X,t}X_{il,t}X_{jl,t}^{⊤}\theta =\bar{X(\theta )}$. On the other hand,

$$\left|\left({\tilde{L}}_{NT}\left({\hat{\delta }}_{ij}\left(x_{l}\right)\right)-{\tilde{L}}_{NT}\left(\delta_{ij}\left(x_{l}\right)\right)\right)X_{il,t}X_{jl,t}^{⊤}\theta \right|\leq 2\frac{\sum_{i<j}\sum_{t}\left[\ell_{NT}\left(\delta_{ij}\left(x_{l}\right)\right)-\ell_{NT}\left({\hat{\delta }}_{ij}\left(x_{l}\right)\right)\right]\bar{X(\theta )}}{\sum_{i<j}\ell_{NT}\left(\delta_{ij}\left(x_{l}\right)\right)}.$$

Now, we note that $\frac{1}{N}\sum_{i<j}\left|\ell_{NT}\left(\delta_{ij}\left(x_{l}\right)\right)-\ell_{NT}\left({\hat{\delta }}_{ij}\left(x_{l}\right)\right)\right|=O_{p}\left({\left(NT\sigma_{n}\right)}^{-1}\right)$ for all l≠i,j, and

(A2) $$\ell_{NT}\left(\delta_{ij}\left(x_{l}\right)\right)=E\left(\ell_{NT}\left(\delta_{ij}\left(x_{l}\right)\right)|X,A,D\right)+\left[\ell_{NT}\left(\delta_{ij}\left(x_{l}\right)\right)-E\left(\ell_{NT}\left(\delta_{ij}\left(x_{l}\right)\right)|X,A,D\right)\right].$$

We note that $E(\ell_{NT}\left(\delta_{ij}\left(x_{l}\right)\right)|X,A,D)$ tends to zero when N→∞. Using Hoeffding’s inequality $\mathrm{Pr}(|\ell_{NT}\left(\delta_{ij}\left(x_{l}\right)\right)-E\left(\ell_{NT}\left(\delta_{ij}\left(x_{l}\right)\right)|X,A,D\right)\left|\;\geq \epsilon_{0}\right)<O\left({\left(NT\right)}^{-1/2}\right)$$\forall \epsilon_{0}>0$. Therefore,

$$\left|\sum_{i<j}\sum_{t}X_{il,t}X_{jl,t}^{⊤}\theta \left({\tilde{L}}_{nt}\left({\hat{\delta }}_{ij}\left(x_{l}\right)\right)\right)-{\tilde{L}}_{nt}\left(\delta_{ij}\left(x_{l}\right)\right)\right|=O_{p}\left({\left(NT\sigma_{n}\right)}^{-1}\right)\;\forall l\neq i,j.$$

Finally, by mean-value expansion for logit distibution Λ, we have

$$\Lambda \left(z_{il,t}\left(\theta ,A_{il}\right)\right)-\Lambda \left(z_{jl,t}\left(\theta ,A_{jl}\right)\right)=\Lambda^{\prime }\left(\eta \right)\left(\sum_{p=1}^{q}\alpha_{p}C_{il,t-p}-\sum_{p=1}^{q}\alpha_{p}C_{jl,t-p}+X_{il,t}X_{jl,t}^{⊤}\theta +A_{ij}\right);$$

then, there exist nonzero $K_{1},K_{2},K_{3}$ such that

$$K_{1}|\Lambda \left(z_{il,t}\left(\theta ,A_{il}\right)\right)-\Lambda \left(z_{jl,t}\left(\theta ,A_{jl}\right)\right)|\leq K_{2}\left|\Lambda \left(z_{il,t}\left(\theta ,A_{il}\right)\right)-\Lambda \left(z_{jl,t}\left(\theta ,A_{jl}\right)\right)\right|,$$

while $\left|{\left(\Lambda^{\prime }\right)}^{-1}\left(\eta \right)\right|\leq K_{3}$. Here,

$$\begin{array}{cc} \left|{\hat{A}}_{l}\left(\theta \right)-A_{l}\left(\theta \right)\right|= & \frac{1}{N}\left|\sum_{i<j}{\tilde{L}}_{NT}\left({\hat{\delta }}_{ij}\left(x_{l}\right)\right)\left(X_{il,t}X_{jl,t}^{⊤}-A_{l}\right)\right|\\ = & \frac{1}{N}|\sum_{i<j}{\tilde{L}}_{NT}\left({\hat{\delta }}_{ij}\left(x_{l}\right)\right)[\Lambda \left(z_{il,t}\left(\theta ,A_{il}\right)\right)-\Lambda \left(z_{jl,t}\left(\theta ,A_{jl}\right)\right)-\Lambda^{\prime }\left(\eta \right)\cdot \\ & \cdot \left(\sum_{p=1}^{q}\alpha_{p}C_{il,t-p}-\sum_{p=1}^{q}\alpha_{p}C_{jl,t-p}\right)]{\left(\Lambda^{\prime }\right)}^{-1}\left(\eta \right)|\\ & \leq \frac{1}{N}|\sum_{i<j}{\tilde{L}}_{NT}\left({\hat{\delta }}_{ij}\left(x_{l}\right)\right)\left[\delta_{ij}\left(x_{l}\right){\left(\Lambda^{\prime }\right)}^{-1}\left(\eta \right)-\left(\sum_{p=1}^{q}\alpha_{p}C_{il,t-p}-\sum_{p=1}^{q}\alpha_{p}C_{jl,t-p}\right)\right]|\\ & \leq \frac{1}{N}|\sum_{i<j}{\tilde{L}}_{NT}\left({\hat{\delta }}_{ij}\left(x_{l}\right)\right)K_{2}K_{3}\delta_{ij}\left(x_{l}\right)|+|\left(\sum_{p=1}^{q}\alpha_{p}C_{il,t-p}-\sum_{p=1}^{q}\alpha_{p}C_{jl,t-p}\right)|\\ & \leq \frac{1}{N}|\sum_{i<j}{\tilde{L}}_{NT}\left({\hat{\delta }}_{ij}\left(x_{l}\right)\right)K_{2}K_{3}\delta_{ij}\left(x_{l}\right)|+O_{p}\left(1\right)\\ & =\frac{1}{N}|\sum_{i<j}\frac{\ell_{NT}\left({\hat{\delta }}_{ij}\left(x_{l}\right)\right)K_{2}K_{3}\delta_{ij}\left(x_{l}\right)}{\sum_{i<j}\ell_{NT}\left({\hat{\delta }}_{ij}\left(x_{l}\right)\left(x_{l}\right)\right)}|+O_{p}\left(1\right)\\ & =O_{p}(\max\left\{{\left(NT\sigma_{N}\right)}^{-1},\sigma_{N}{\left(NT\right)}^{-1}\right\}), \end{array}$$

where in the last equality we use Equation (A2). □

**Proof** **of** **Theorem** **5.** By Theorem 2 (Law of Large Numbers) and Theorem 3, we have

Ea(H^(C))=−∑i<j∑t=1TEa(lij,tθ^,a^Nlog(lij,tθ^,a^N)|F)→Pr−TN2−1∑i<jElogzij,t(θ0,aN0)1+zij,t(θ0,aN0)log(lij,tθ0,aN0)=−T∑i<jE(lij,tθ0,aN0log(lij,tθ0,aN0))=−∑i<j∑t=1Tlij,tθ0,aN0log(lij,tθ0,aN0)=H0(C).

Then, for all ϵ>0, $|\hat{H}\left(C\right)-H_{0}\left(C\right)|<\epsilon$. Let us set $\epsilon =\frac{1}{NT}\sqrt{\log(NT)}$; then,

(A3) $$\mathrm{Pr}\left(\left|\hat{H}\left(C\right)-H_{0}\left(C\right)\right|\geq \frac{1}{NT}\sqrt{\log(NT)}\right)\leq \frac{\log(NT)}{{\left(\frac{1}{NT}\sqrt{\log(NT)}\right)}^{2}NT}=O\left({\left(NT\right)}^{-1}\right).$$

Hence, with probability of at least $1-O({\left(NT\right)}^{-1})$, we have $\left|\hat{H}\left(C\right)-H_{0}\left(C\right)\right|<\frac{1}{NT}$$\sqrt{\log(NT)}$ for any network *C* containing the pair of nodes i,j. Taking the supremum over all C∈C, we have

(A4) $$\underset{C\in C}{\sup}|\hat{H}\left(C\right)-H_{0}\left(C\right)|<\frac{1}{NT}\sqrt{\log(NT)},$$

with probability $1-O({\left(NT\right)}^{-1})$. □

**Proof** **of** **Theorem** **6.** From Theorem 3 and the first-order condition associated with the concentrated log-likelihood, a mean value expansion offers

$$\sqrt{NT}\left(\hat{\theta }-\theta^{0}\right)=-{\left[\frac{1}{NT}\sum_{i<j}\sum_{t=1}^{T}\frac{\partial s_{ijt,\theta }\left(\bar{\theta },{\hat{a}}_{N}\left(\bar{\theta }\right)\right)}{\partial \theta }\right]}^{-1}\left[\frac{1}{\sqrt{NT}}\sum_{j<i}\sum_{t=1}^{T}\frac{\partial s_{ijt,\theta }\left(\theta^{0},{\hat{a}}_{N}\left(\theta^{0}\right)\right)}{\partial \theta }\right],$$

where $s_{ijt,\theta }\left(\theta ,a_{N}\right)$ denotes the {ij}th dyad’s contributions to the score of the maximum likelihood estimator associated with vector θ. After applying the result for the Hessian of the concentrated log-likelihood derived immediately above, we obtain

(A5) $$\sqrt{NT}\left(\hat{\theta }-\theta^{0}\right)=J_{0}^{-1}\left(\theta \right)\times \left[\frac{1}{\sqrt{NT}}\sum_{j<i}\sum_{t=1}^{T}\frac{\partial E\left(s_{ijt,\theta }\left(\theta^{0},{\hat{a}}_{N}\left(\theta^{0}\right)\right)\right)}{\partial \theta }\right]+o_{p}\left(1\right)$$

since $\frac{1}{NT}\sum_{i<j}\sum_{t=1}^{T}\frac{\partial E\left(s_{ijt,\theta }\left(\bar{\theta },{\hat{a}}_{N}\left(\bar{\theta }\right)\right)\right)}{\partial \theta }\overset{\mathrm{Pr}}{\to }-J_{0}\left(\theta \right)$. Tedious calculations, along with the calculations immediately above, produce

(A6) $$\frac{1}{\sqrt{NT}}\sum_{j<i}\sum_{t=1}^{T}\frac{\partial E\left(s_{ijt,\theta^{0}}\left(\theta^{0},{\hat{a}}_{N}\left(\theta^{0}\right)\right)\right)}{\partial \theta }=\frac{1}{\sqrt{NT}}\sum_{j<i}\sum_{t=1}^{T}\frac{\partial E\left({\hat{s}}_{ijt,\theta^{0}}\left(\theta^{0},{\hat{a}}_{N}^{0}\right)\right)}{\partial \theta }+o_{p}\left(1\right),$$

where ${\hat{s}}_{ijt,\hat{\theta }}\left(\hat{\theta },{\hat{a}}_{N}\right)=s_{ijt,\hat{\theta }}\left(\hat{\theta },{\hat{a}}_{N}\right)-H_{\theta a_{N}^{⊤}}\left(C\right)H_{a_{N}a_{N}^{⊤}}\left(C\right)\cdot s_{ijt,a_{N}}\left(\hat{\theta },{\hat{a}}_{N}\right)$. Substituting Equation (A6) into Equation (A5) results in

(A7) $$\sqrt{NT}\left(\hat{\theta }-\theta^{0}\right)=J_{0}^{-1}\left(\theta \right)+J_{0}^{-1}\left(\theta \right)\frac{1}{\sqrt{NT}}\sum_{j<i}\sum_{t=1}^{T}\frac{\partial E\left({\hat{s}}_{ijt,\theta^{0}}\left(\theta^{0},{\hat{a}}_{N}\right)\right)}{\partial \theta }+o_{p}\left(1\right)$$

Applying the Central Limit Theorem to the second addend, we have $\sqrt{NT}\left(\hat{\theta }-\theta^{0}\right)\overset{D}{\to }N\left(J_{0}^{-1}\left(\theta \right),J_{0}{\left(\theta \right)}^{1/2}\right)$. □

**Proof** **of** **Theorem** **7.** (i) Here, we note that

$$\begin{array}{cc} H(C)= & -\sum_{i<j}\sum_{t=1}^{T}\frac{l_{ij,t}^{\theta ,a_{N}}}{K}\log\left(\frac{l_{ij,t}^{\theta ,a_{N}}}{K}\right)=-\sum_{i=1}^{N-1}\sum_{j=i+1}^{N}\sum_{t=1}^{T}\frac{l_{ij,t}^{\theta ,a_{N}}}{K}\log\left(\frac{l_{ij,t}^{\theta ,a_{N}}}{K}\right)\\ & =-\sum_{t=1}^{T}\left[\sum_{i=1}^{N-1}\sum_{j=i+2}^{N-1}\frac{l_{ij,t}^{\theta ,a_{N}}}{K}\log\left(\frac{l_{ij,t}^{\theta ,a_{N}}}{K}\right)-\sum_{i=1}^{N-1}\frac{l_{i(i+1),t}^{\theta ,a_{N}}}{K}\log\left(\frac{l_{i(i+1),t}^{\theta ,a_{N}}}{K}\right)\right.\\ & \left.-\sum_{i=1}^{N-1}\frac{l_{iN,t}^{\theta ,a_{N}}}{K}\log\left(\frac{l_{iN,t}^{\theta ,a_{N}}}{K}\right)\right], \end{array}$$

where we can write

$$\sum_{i=1}^{N-1}\sum_{j=i+2}^{N-1}l_{ij,t}^{\theta ,a_{N}}=\sum_{i=1}^{N-1}\sum_{j=i+1}^{N}l_{ij,t}^{\theta ,a_{N}}-\sum_{i=1}^{N-1}\left(l_{i,i+1}^{\theta ,a_{N}}-l_{iN}^{\theta ,a_{N}}\right),$$

allowing us definition of a new vector over the parameters, i.e., $q_{t}={\left(q_{ij,t}\right)}_{i=1,j=i+1}^{N-1,N}$ as

$$q_{ij,t}=\frac{l_{ij,t}^{\theta ,a_{N}}}{\sum_{k=1}^{N-1}\sum_{l=k+1}^{N}l_{kl,t}-\sum_{k=1}^{N-1}\left(l_{k(k+1),t}^{\theta ,a_{N}}-l_{kN,t}^{\theta ,a_{N}}\right)}=\frac{l_{ij,t}^{\theta ,a_{N}}}{\rho_{kl,N}}.$$

From this definition arises the fact that $0\leq q_{ij,t}\leq 1$; then,

$$\begin{array}{cc} H(q_{t})= & -\sum_{i=1}^{N-1}\sum_{j=i+1}^{N}q_{ij,t}\log\left(q_{ij,t}\right)\\ & =-\sum_{i=1}^{N-1}\sum_{j=i+2}^{N-1}\frac{l_{ij,t}^{\theta ,a_{N}}}{\rho_{kl,N}}\log\left(\frac{l_{ij,t}^{\theta ,a_{N}}}{\rho_{kl,N}}\right)\\ & =-\sum_{i=1}^{N-1}\sum_{j=i+2}^{N-1}\frac{l_{ij,t}^{\theta ,a_{N}}}{\rho_{kl,N}}\left[\log\left(\frac{l_{ij,t}^{\theta ,a_{N}}}{K}\right)+\log\left(\frac{K}{\rho_{kl,N}}\right)\right]\\ & =-\frac{K}{\rho_{kl,N}}\sum_{i=1}^{N-1}\sum_{j=i+2}^{N-1}\frac{l_{ij,t}^{\theta ,a_{N}}}{K}\log\left(\frac{l_{ij,t}^{\theta ,a_{N}}}{K}\right)-\sum_{i=1}^{N-1}\sum_{j=i+2}^{N}\frac{l_{ij,t}^{\theta ,a_{N}}}{\rho_{kl,N}}\log\left(\frac{K}{\rho_{kl,N}}\right)\\ & =-\frac{K}{\rho_{kl,N}}\sum_{i=1}^{N-1}\sum_{j=i+2}^{N-1}\frac{l_{ij,t}^{\theta ,a_{N}}}{K}\log\left(\frac{l_{ij,t}^{\theta ,a_{N}}}{K}\right)-\log\left(\frac{K}{\rho_{kl,N}}\right). \end{array}$$

Returning to the entropy with respect to vector $q_{t}$, we have

$$-\sum_{i=1}^{N-1}\sum_{j=i+2}^{N-1}\frac{l_{ij,t}^{\theta ,a_{N}}}{K}\log\left(\frac{l_{ij,t}^{\theta ,a_{N}}}{K}\right)=\frac{\rho_{kl,N}}{K}\left[H\left(q_{t}\right)+\log\left(\frac{K}{\rho_{kl,N}}\right)\right],$$

and via Theorem 2.6.4 from [51],

$$H\left(q_{t}\right)\leq \log\left(N\left(N-1\right)\right).$$

Then,

$$-\sum_{i=1}^{N-1}\sum_{j=i+2}^{N-1}\frac{l_{ij,t}^{\theta ,a_{N}}}{K}\log\left(\frac{l_{ij,t}^{\theta ,a_{N}}}{K}\right)\leq \frac{\rho_{kl,N}}{K}\log\left(\frac{K(N(N-1))}{\rho_{kl,N}}\right).$$

Therefore, the entropy satisfies

$$\begin{array}{cc} H(C) & =-\sum_{t=1}^{T}\left[\sum_{i=1}^{N-1}\sum_{j=i+2}^{N-1}\frac{l_{ij,t}^{\theta ,a_{N}}}{K}\log\left(\frac{l_{ij,t}^{\theta ,a_{N}}}{K}\right)-\sum_{i=1}^{N-1}\frac{l_{i(i+1),t}^{\theta ,a_{N}}}{K}\log\left(\frac{l_{i(i+1),t}^{\theta ,a_{N}}}{K}\right)\right.\\ & \left.-\sum_{i=1}^{N-1}\frac{l_{iN,t}^{\theta ,a_{N}}}{K}\log\left(\frac{l_{iN,t}^{\theta ,a_{N}}}{K}\right)\right]\\ & \leq \frac{\rho_{kl,N}}{K}\log\left(\frac{K(N(N-1))}{\rho_{kl,N}}\right)-\sum_{i=1}^{N-1}\frac{l_{i(i+1),t}^{\theta ,a_{N}}}{K}\log\left(\frac{l_{i(i+1),t}^{\theta ,a_{N}}}{K}\right)\\ & -\sum_{i=1}^{N-1}\frac{l_{iN,t}^{\theta ,a_{N}}}{K}\log\left(\frac{l_{iN,t}^{\theta ,a_{N}}}{K}\right) \end{array}$$

$$\begin{array}{cc} & \leq \frac{\rho_{kl,N}}{K}\log\left(\frac{K(N(N-1))}{\rho_{kl,N}}\right)-\frac{1}{K}[\left(\sum_{i=1}^{N-1}{\hat{p}}_{i,i+1}\right)\log\left(\frac{\sum_{i=1}^{N-1}l_{i(i+1),t}^{\theta ,a_{N}}}{(N-1)K}\right)-\\ & -\left(\sum_{i=1}^{N-1}l_{iN,t}^{\theta ,a_{N}}\right)\log\left(\frac{\sum_{i=1}^{N-1}l_{iN,t}^{\theta ,a_{N}}}{(N-1)k}\right)]. \end{array}$$

(ii) This proof is based on the fact that logarithms are concave functions. We know that $\log_{b}\left(x\right)-\log_{b}\left(y\right)\leq \frac{1}{\ln(b)}\left(\frac{x-y}{y}\right)$, ∀x,y>0. Then,

$$\log\left(\varrho_{ij,F}\right)-\log\left(\varrho_{ij,F^{\prime }}\right)\leq \frac{1}{\ln(2)}\left(\frac{\varrho_{ij,F}-\varrho_{ij,F^{\prime }}}{\varrho_{ij,F^{\prime }}}\right),$$

allowing us constructioon of the following inequality:

$$\varrho_{ij,F}\log\left(\varrho_{ij,F}\right)-\varrho_{ij,F}\log\left(\varrho_{ij,F^{\prime }}\right)\leq \frac{1}{\ln(2)}\left(\frac{{\left(\varrho_{ij,F}\right)}^{2}-\varrho_{ij,F}\varrho_{ij,F^{\prime }}}{\varrho_{ij,F^{\prime }}}\right).$$

Then,

$$-\varrho_{ij,F}\log\left(\varrho_{ij,F}\right)\geq -\frac{1}{\ln(2)}\left(\frac{{\left(\varrho_{ij,F}\right)}^{2}-\varrho_{ij,F}\varrho_{ij,F^{\prime }}}{\varrho_{ij,F^{\prime }}}\right)-\varrho_{ij,F}\log\left(\varrho_{ij,F^{\prime }}\right).$$

Finally, considering the sum over *i* and over the corresponding *j*, we have the desired result. □

### Appendix A.2. Weighted Likelihood

We start by investigating a panel data logit model with fixed effects, a *p*-lagged dependent variable, and a set of strictly exogenous explanatory variables. We let

$$C_{ij}^{T}=\left(C_{ij,t-1},C_{ij,t-2},\ldots \right).$$

The model logit can be written as

(A8) $$\mathrm{Pr}\left(C_{ij,t}=1|C^{T-1},X,D,A\right)=\frac{\exp\left(\sum_{l=1}^{p}\alpha_{l}C_{ij,t-l}+\beta X_{ij,t}^{⊤}+D_{ij,t}+A_{ij}\right)}{1+\exp\left(\sum_{l=1}^{p}\alpha_{l}C_{ij,t-l}+\beta X_{ij,t}^{⊤}+D_{ij,t}+A_{ij}\right)},$$

with i,j∈{1,…,N} and t∈{1,…,T}. We assume that the autoregressive order p∈{1,2,…} is known, and the outcomes $C_{ij,t}$ are observed for time $t=t_{0},\ldots ,T$. The total number of time periods for which outcomes are observed is $T_{\mathrm{obs}}=T+p$. All probabilistic statements are for the model distribution generated under the true model parameters $\alpha^{0}$ and $\beta^{0}$. For example, if $C_{ij}={\left(C_{ij,1},\ldots ,C_{ij,T}\right)}^{⊤}$, $X_{ij}={\left(X_{ij,1},\ldots ,X_{ij,T}\right)}^{⊤}$, and $D_{ij}={\left(D_{ij,1},\ldots ,D_{ij,T}\right)}^{⊤}$, then $\mathrm{Pr}\left(C_{ij,t}=1|C_{ij,0}=c_{0},X_{ij}=x,D_{ij}=d,A_{ij}=a\right)=p_{c_{0}}\left(\alpha^{0},\beta^{0},c,x,d,a\right)$, where

(A9) $$\begin{array}{ccc} p_{c_{0}}\left(\alpha ,\beta ,c,x,d,a\right) & := & \prod_{i<j}\prod_{t=1}^{T}{\left[\frac{1}{1+\exp\left(\sum_{l=1}^{p}\alpha_{l}c_{ij,t-l}+\beta x_{ij,t}^{⊤}+d_{ij,t}+a_{ij}\right)}\right]}^{1-c_{ij,t}}\\ & \times & {\left[\frac{\exp\left(\sum_{l=1}^{p}\alpha_{l}c_{ij,t-l}+\beta x_{ij,t}^{⊤}+d_{ij,t}+a_{ij}\right)}{1+\exp\left(\sum_{l=1}^{p}\alpha_{l}c_{ij,t-l}+\beta x_{ij,t}^{⊤}+d_{ij,t}+a_{ij}\right)}\right]}^{c_{ij,t}}. \end{array}$$

We drop index ij until we discuss estimation, that is, instead of $C_{ij,t}$, $X_{ij,t}$, $D_{ij,t}$$A_{ij}$, we just write *C* , *X*, *D*, and *A* for the corresponding random variables. Our first goal is to obtain the conditional likelihood under some considerations in Equation (1) that are valid regardless of the realization of *A* fixed effects. Following [44], we consider events

$$\begin{array}{c} B_{1}=\{c_{ij,0}=c_{0},\ldots ,c_{ij,t-1}=c_{t-1},c_{ij,t}=0,c_{ij,t+1}=c_{t+1},\ldots ,\\ c_{ij,s-1}=c_{s-1},c_{ij,s}=1,c_{ij,s+1}=c_{s+1},\ldots ,c_{ij,T}=c_{T}\}, \end{array}$$

$$\begin{array}{c} B_{2}=\{c_{ij,0}=c_{0},\ldots ,c_{ij,t-1}=c_{t-1},c_{ij,t}=1,c_{ij,t+1}=c_{t+1},\ldots ,\\ c_{ij,s-1}=c_{s-1},c_{ij,s}=0,c_{ij,s+1}=c_{s+1},\ldots ,c_{ij,T}=c_{T}\}, \end{array}$$

where 1≤t<s<T−1 and $c_{0},c_{1},c_{2},\ldots ,c_{T}$ are either 0 or 1. A simple calculation shows that

$$\begin{array}{ccc} \mathrm{Pr}(B_{1}|c,x,d,a) & = & \frac{1}{1+\exp\left(\sum_{l=1}^{p}\alpha_{l}c_{t-l}+\beta x_{ij,t}^{⊤}+d_{ij,t}+a_{ij}\right)}\\ & \times & \frac{\exp{\left(\sum_{l=2}^{p}\alpha_{l}c_{t-l}+\beta x_{ij,t}^{⊤}+d_{ij,t}+a_{ij}\right)}^{c_{t+1}}}{1+\exp\left(\sum_{l=2}^{p}\alpha_{l}c_{t-l}+\beta x_{ij,t}^{⊤}+d_{ij,t}+a_{ij}\right)}\\ & \times & \frac{\exp\left(\sum_{l=1}^{p}\alpha_{l}c_{s-l}+\beta x_{ij,s}^{⊤}+d_{ij,s}+a_{ij}\right)}{1+\exp\left(\sum_{l=1}^{p}\alpha_{l}c_{s-l}+\beta x_{ij,s}^{⊤}+d_{ij,s}+a_{ij}\right)}\\ & \times & \frac{\exp{\left(\sum_{l=1}^{p}\beta_{l}c_{s-l}+\beta x_{ij,s+1}^{⊤}+d_{ij,s+1}+a_{ij}\right)}^{c_{s+1}}}{1+\exp\left(\sum_{l=1}^{p}\alpha_{l}c_{s-l}+\beta x_{ij,s+1}^{⊤}+d_{ij,s+1}+a_{ij}\right)}, \end{array}$$

and

$$\begin{array}{ccc} \mathrm{Pr}(B_{2}|c,x,d,a) & = & \frac{\exp\left(\sum_{l=1}^{p}\alpha_{l}c_{t-l}+\beta x_{ij,t}^{⊤}+d_{ij,t}+a_{ij}\right)}{1+\exp(\sum_{l=1}^{p}\alpha_{l}c_{t-l}+\beta x_{ij,t}^{⊤}+d_{ij,t}+a_{ij})}\\ & \times & \frac{\exp{\left(\sum_{l=1}^{p}\alpha_{l}c_{t-l}+\beta x_{ij,t}^{⊤}+d_{ij,t}+a_{ij}\right)}^{c_{t+1}}}{1+\exp\left(\sum_{l=1}^{p}\alpha_{l}c_{t-l}+\beta x_{ij,t}^{⊤}+d_{ij,t}+a_{ij}\right)}\\ & \times & \frac{1}{1+\exp\left(\sum_{l=1}^{p}\alpha_{l}c_{s-l}+\beta x_{ij,s}^{⊤}+d_{ij,s}+a_{ij}\right)}\\ & \times & \frac{\exp{\left(\sum_{l=2}^{p}\alpha_{l}c_{s-l}+\beta x_{ij,s+1}^{⊤}+d_{ij,s+1}+a_{ij}\right)}^{c_{s+1}}}{1+\exp\left(\sum_{l=2}^{p}\alpha_{l}c_{s-l}+\beta x_{ij,s+1}^{⊤}+d_{ij,s+1}+a_{ij}\right)}. \end{array}$$

Then,

(A10) $$\begin{array}{c} \mathrm{Pr}(B_{1}|c,x,d,a,B_{1}\cup B_{2},x_{ij,t+1}=x_{ij,s+1})=\\ \frac{1}{1+\exp\left(\left(x_{t}-x_{s}\right)\beta +\alpha_{1}\left(c_{t+1}-c_{s+1}\right)+\sum_{l=1}^{p}\alpha_{l}\left(c_{t-l}-c_{s-l}\right)1\left\{s-t>1\right\}\right)}, \end{array}$$

and

(A11) $$\begin{array}{c} \mathrm{Pr}(B_{2}|c,x,d,a,B_{1}\cup B_{2},x_{ij,t+1}=x_{ij,s+1})=\\ \frac{\exp\left(\left(x_{t}-x_{s}\right)\beta +\alpha_{1}\left(c_{t+1}-c_{s+1}\right)+\sum_{l=1}^{p}\alpha_{l}\left(c_{t-l}-c_{s-l}\right)1\left\{s-t>1\right\}\right)}{1+\exp\left(\left(x_{t}-x_{s}\right)\beta +\alpha_{1}\left(c_{t+1}-c_{s+1}\right)+\sum_{l=1}^{p}\alpha_{l}\left(c_{t-l}-c_{s-l}\right)1\left\{s-t>1\right\}\right)}, \end{array}$$

which does not depend on $a_{ij}$. In the special case where all the explanatory variables and the stochastic process ${\left\{x_{ij,t}\right\}}_{t\in T}$ satisfy $\mathrm{Pr}(x_{ij,t+1}-x_{ij,s+1}=0)>0$, we can use Equations (A10) and (A11) to make inference about α and β. In particular,

(A12) (α^,β^)=argmaxα,β1TN2−1∑i<j∑2≤t<s≤T−11{cij,t+cij,s=1}Kxij,t+1−xij,s+1σn×logexp(xt−xs)b+a1(ct+1−cs+1)+∑l=1pal(ct−l−cs−l)1{s−t>1}cij,t1+exp(xt−xs)b+a1(ct+1−cs+1)+∑l=1pbl(ct−l−cs−l)1{s−t>1}.

Here, K(·) is a kernel density function that gives appropriate weight to link {ij}, while $\sigma_{n}$ is a bandwidth that shrinks as *n* increases. The asymptotic theory requires that kernel density be chosen so that a number of regularity conditions cn be determined. If $\mathrm{Pr}(X_{ij,t+1}=X_{ij,s+1})>0$ (e.g., discrete covariates or controlled experiments) and $X_{ij,t+1}-X_{ij,s+1}$ has sufficient variation conditional on $X_{ij,t+1}=X_{ij,s+1}$, then the K(·) function can be replaced by a $1(X_{ij,t+1}-X_{ij,s+1}=0)$ indicator function, and the resulting estimator has the usual ${\left(NT\right)}^{-1/2}$ rate of convergence. However, if the regressors are continuous or have high dimensions, then the estimator, while still consistent and asymptotically normal, has a convergence rate slower than ${\left(NT\right)}^{-1/2}$. Also, this rate falls as the number of covariates increases.
